# Genome-wide identification and characterization of gene family for RWP-RK transcription factors in wheat (*Triticum aestivum* L.)

**DOI:** 10.1371/journal.pone.0208409

**Published:** 2018-12-12

**Authors:** Anuj Kumar, Ritu Batra, Vijay Gahlaut, Tinku Gautam, Sanjay Kumar, Mansi Sharma, Sandhya Tyagi, Krishna Pal Singh, Harindra Singh Balyan, Renu Pandey, Pushpendra Kumar Gupta

**Affiliations:** 1 Advance Center for Computational & Applied Biotechnology, Uttarakhand Council for Biotechnology (UCB), Dehradun, India; 2 Department of Genetics and Plant Breeding, CCS University, Meerut, India; 3 Department of Plant Molecular Biology, South Campus, University of Delhi, Delhi, India; 4 Bioinformatics Centre, Biotech Park, Lucknow, India; 5 ICMR- National Institute of Cancer Prevention and Research, Noida, India; 6 Division of Plant Physiology, ICAR-Indian Agricultural Research Institute, New Delhi, India; 7 Ch. Charan Singh Haryana Agricultural University, Hisar, India; National Institute of Plant Genome Research, INDIA

## Abstract

RWP-RKs represent a small family of transcription factors (TFs) that are unique to plants and function particularly under conditions of nitrogen starvation. These RWP-RKs have been classified in two sub-families, NLPs (NIN-like proteins) and RKDs (RWP-RK domain proteins). NLPs regulate tissue-specific expression of genes involved in nitrogen use efficiency (NUE) and RKDs regulate expression of genes involved in gametogenesis/embryogenesis. During the present study, using *in silico* approach, 37 wheat *RWP-RK* genes were identified, which included 18 *TaNLPs* (2865 to 7340 bp with 4/5 exons), distributed on 15 chromosomes from 5 homoeologous groups (with two genes each on 4B,4D and 5A) and 19 *TaRKD*s (1064 to 5768 bp with 1 to 6 exons) distributed on 12 chromosomes from 4 homoeologous groups (except groups 1, 4 and 5); 2–3 splice variants were also available in 9 of the 37 genes. Sixteen (16) of these genes also carried 24 SSRs (simple sequence repeats), while 11 genes had targets for 13 different miRNAs. At the protein level, MD simulation analysis suggested their interaction with nitrate-ions. Significant differences were observed in the expression of only two (*TaNLP1* and *TaNLP*2) of the nine representative genes that were used for *in silico* expression analysis under varying levels of N at post-anthesis stage (data for other genes was not available for *in silico* expression analysis). Differences in expression were also observed during qRT-PCR, when expression of four representative genes (*TaNLP2*, *TaNLP7*, *TaRKD6* and *TaRKD9*) was examined in roots and shoots of seedlings (under different conditions of N supply) in two contrasting genotypes which differed in NUE (C306 with low NUE and HUW468 with high NUE). These four genes for qRT-PCR were selected on the basis of previous literature, level of homology and the level of expression (*in silico* study). In particular, the *TaNLP7* gene showed significant up-regulation in the roots and shoots of HUW468 (with higher NUE) during N-starvation; this gene has already been characterized in Arabidopsis and tobacco, and is known to be involved in nitrate-signal transduction pathway.

## Introduction

Nitrogen (N) is an essential element for plant growth, productivity and grain quality. It plays a vital role in various metabolic activities within the cell involving synthesis of a variety of macromolecules, such as nucleic acids, proteins, cofactors, chlorophyll and other molecules involved in signaling and storage [1,2]. Only 30% of available soil N is taken up by plant roots in the form of nitrate (NO_3_^−^) and ammonium (NH_4_^+^) ions, amino acids and other organic molecules. The remaining 70% N is lost either through leaching into the soil or in the gaseous form into the atmosphere, the latter also causing environmental pollution. Therefore, efforts are being made to improve N use efficiency (NUE) of crop plants so that high yielding crops can be grown with low N-input without significant yield loss [3].

The NUE includes two major components, N uptake efficiency (NUpE) and N utilization (assimilation) efficiency (NUtE), although N transport and N remobilization (after assimilation) are two other components. Each of these components is controlled by a number of genes [2] including a family of TFs called RWP-RKs, so named due to the presence of a conserved RWP-RK motif [4]. These RWP-RK genes are ubiquitous in plants and have been classified in two sub-families, NLPs (NIN-like proteins) and RKDs (RWP-RK domain proteins). NLPs regulate tissue-specific expression of genes involved in nitrogen use efficiency (NUE) [4–7] and RKDs regulate expression of genes involved in gametogenesis/embryogenesis [4,8]. The RWP-RK proteins bind to *cis*-acting elements in the promoter regions of NUE-related genes [including nitrate reductase (*NR/NIA1*) and nitrite reductase (*NiR1*)] and the genes responsible for gametogenesis and embryogenesis [9]. Both NLPs and RKDs contain a common but dissimilar DNA binding RWP-RK domain [4,9], but NLPs also carry an additional domain called Phox/Bem1 (PB1), which is an octicosapeptide that allows interactions with additional proteins [4,5]. The N-terminal regions of NLPs respond to nitrate signals and bind specifically to the nitrate responsive elements (NREs) that are found in the promoter regions of nitrate-inducible gene loci [5,9,10].

The function of NLPs under conditions of different NO_3_^−^ ion concentrations has been investigated in a number of plant species including Arabidopsis (*A*. *thaliana*), rice (*Oryza sativa*) and tobacco (*Nicotiana tabacum*) [4,6,9]. These studies demonstrated that NLPs control expression of nitrate inducible genes including those associated with NUE [4–7], and RKDs take part in N signaling [8]. It has been shown that the activity of NLPs is post-translationally modulated by nitrate signaling, so that the suppression of NLPs impairs the nitrate-inducible expression of various genes causing severe growth inhibition [6]. In non-nodulating plants, the most-studied member of RWP-RK family is *NLP7*, which is involved in nitrate-signal transduction pathway, thus regulating N assimilation [10].

In wheat (*Triticum aestivum*), the RWP-RK genes have never been subjected to a detailed study. During the present study, we identified and conducted a comprehensive study of 37 wheat RWP-RK genes (19 *TaRKDs* and 18 *TaNLPs*) and their encoded proteins. These genes were subjected to a systematic *in silico* analysis to study the gene structure and promoter sequences; the corresponding proteins were examined for the functional domains, conserved motifs, physicochemical properties, homology modeling, molecular docking, molecular dynamics (MD) simulations and phylogenetic relationships. The study also included *in silico* expression of these genes in different tissues at different developmental stages under varying levels of N supply. Gene ontology (GO) analysis was also conducted to determine the functions of these genes. Four representative genes (*TaRKD6*, *TaRKD9*, *TaNLP2*, *TaNLP7*) that were selected on the basis of level of expression (*in silico*), previous literature and level of homology, were also used for qRT-PCR using two contrasting wheat genotypes (C306 and HUW468), which differ for NUE. The study provides an insight into the family of wheat genes encoding RWP-RK TFs, both at gene and protein levels.

## Materials and methods

### Analysis of *RWP-RK* genes

#### Identification and analysis

In order to obtain wheat gene sequences that encode RWP-RK TFs, sequences of 18 known *RWP-RK* genes from the model species *Brachypodium distachyon* (a closely related species to wheat) were used. Coding DNA sequences (CDSs) of *B*. *distachyon* genes were retrieved from Ensembl Plants (http://plants.ensembl.org/) and used in tBLASTx analysis against the recently released wheat genome assembly (IWGSC RefSeq v1.0) available on Ensembl Plants. HMMER tool (available at Ensembl Plants) was also used to retrieve additional genes. Following criteria were used for the identification of wheat orthologs [11–13]: (*i*) high level (>60%) of sequence identity and query coverage along the protein length; (*ii*) presence of all the domains and motifs available in query sequences. Homoeologous relationships between genes were established on the basis of their chromosome assignment and percentage of protein sequence identity (>90%).

Analysis of the structure of wheat RWP-RK genes was conducted using Gene Structure Display Server (GSDS) v2.0 by comparing their full length CDSs with their corresponding genomic sequences [14]. Intron phases (phase 0, 1, 2) were identified following the criteria used by us earlier [13]. MapInspect (http://www.plantbreeding.wur.nl/UK/software_map-inspect.html) was used to physically map the genes onto individual wheat chromosomes. MCScanX was used to predict the segmental/tandem gene duplications. The values of synonymous (Ks) and non-synonymous (Ka) substitutions were obtained using CDS of wheat genes with respect to CDS of the corresponding Brachypodium genes. One kb genomic region upstream of the translation start site (ATG) (i.e. promoter region) of each gene was analysed for the presence of cis-regulatory response elements using PlantCARE database [15]. Only the response elements on the sense strand showing a matrix value of ≥5 were accepted. Simple sequence repeats (SSRs) were identified within the gene sequences using BatchPrimer3v1.0 (http://probes.pw.usda.gov/batchprimer3/). The miRNAs and their targets in RWP-RK genes were predicted employing web-based psRNATarget server [16] using default parameters.

#### Synteny and collinearity analyses

Synteny/collinearity analysis was conducted by comparing a block of 21 genes associated with *TaRKD* and *TaNLP* genes (10 genes on either side of the gene of interest) with Brachypodium, rice and sorghum employing URGI database (http://wheat-urgi.versailles.inra,fr/) The results of the above analysis were visualized using the program circos version 0.67 [17].

### *In silico* gene expression profiling and hierarchical clustering

*In silico* gene expression profiling was based on the assumption that homoeologous genes shared common probe ids. Based on this property, we selected one candidate probe id for each group of homoeologous genes. The selected probe ids were retrieved from the “Affymetrix wheat 61K microarray” platform using the PLEXdb interface [18]. The expression analysis was carried out for the following genes using microarray data in Genevestigator platform [19]: (*i*) 9 *TaRKD* (from 3 homoeologous groups) and 18 *TaNLP* genes (from 6 homoeologous groups) in 15 different tissues, 10 developmental stages and in leaves under varying levels of N availability at post-anthesis, and (ii) wheat orthologs of 4 downstream genes (including *4CL1*, *CHX17*, *SIR* and *AMP* dependent synthetase) induced by *AtNLP7* in Arabidopsis under varying levels of N. The expression profiles of genes identified from wheat microarray data were used for construction of the heat map using hierarchical clustering tool embedded in Genevestigator platform.

### Analysis of RWP-RK protein sequences

Major domains in the predicted protein sequences of wheat genes belonging to RWP-RK family were identified through conserved domain (CD)-search program of conserved domain database (CDD) at NCBI. Manual search was done for RWPXRK domain, which is known to be a characteristic feature of RWP-RK TF family in plants [4]. Physicochemical properties of all the predicted proteins were studied using ProtParam server (https://web.expasy.org/protparam/). The sub-cellular location of wheat genes was predicted using a user-friendly web-server PLANT-mPLoc [20].

#### 3D structure, its evaluation and optimization

The three dimensional (3D) structures of predicted proteins were deduced using homology modeling [21]. For this purpose, PSI-BLAST was first carried out against protein data bank (PDB) (http://www.rcsb.org/pdb/home/home.do) and swissProt template library (STL) (http://swissmodel.expasy.org/workspace/index.php?func = tools_smtl) to find out the suitable homologous templates (crystal and NMR 3D structures) on the basis of maximum identity, maximum score and minimum e-value. The best template for each gene from PSI-BLAST was selected in SwissModel server to generate the 3D structures of the predicted proteins [22]. The geometric evaluation of the predicted 3D structures of proteins for different genes was performed using PROCHECK and protein structure verification server (PSVS) (htttp://nihserver.mbi.ucla. edu/SAVES/). Ramachandran plots were prepared through calculation of phi (Φ) and psi (ψ) torsion angles.

#### Alignment of 3D structures of predicted wheat proteins over 3D structures of query proteins

To check the correct topology of 3D structures of predicted proteins of wheat genes, the generated structures of encoded proteins predicted for one representative gene each belonging to RKD and NLP sub-families of *RWP-RK* genes were aligned with the 3D structures of proteins encoded by respective query genes belonging to Brachypodium using FATCAT server [23]. The similarity of the generated 3D structures in a globally optimized superimposition environment was measured by comparing RMSD value of the Cα atoms of the generated structures to those of the corresponding 3D structures of the query genes. The function of RWP-RK (as TFs) at the biochemical level was predicted from the 3D structures of proteins using ProFunc server [24].

#### Molecular docking of 3D structures of the predicted wheat proteins with nitrate ions

The predicted 3D protein structures of representative wheat proteins TaRKD6-2A and TaNLP7-3A were used for docking studies, since these proteins showed maximum amino acids in the favoured regions in Ramachandran plots (S5 Table). Structure of nitrate ion (CID_94310) was retrieved from PubChem database in SDF format. To find out the interacting residues between 3D protein structures and nitrate ion, docking studies were performed by Surflex Dock program available in SYBYL-X (https://www.certara.com/software/molecular-modeling-and-simulation/sybyl-x-suite/). For docking analysis, hydrogen atoms were added to the predicted 3D protein structures (TaRKD6-2A and TaNLP7-3A) using AMBER7 FF99 charges. The 3D structures, thus obtained, were optimized by applying 100 steps of Powell method and Conjugate gradient algorithm with Tripos force field. Grid generation was done by automated mode of Protomol tool on active regions of both 3D protein structures. A maximum of 20 conformations of nitrate-ion with both the 3D protein structures were generated. On the basis of scoring function, top scoring conformations were selected for protein stability through MD simulations [25,26].

#### MD simulations

The MD simulation analysis was conducted using the Desmond v4.8 suite available in Schrodinger-Maestro v11. Energy minimization (100 steps steepest descent followed by 2000 steps conjugate gradient) was undertaken before initiating the MD simulation to remove initial steric clashes. Fourteen (14) Na^+^ ions were added to each 3D structure and nitrate ion complex with salt atoms that maintain the system at charge neutrality. The system was then solvated using SPC water molecules within Triclinic box in a periodic box condition. Both 3D protein and nitrate complexes had at least 5Å buffer in every direction of the box to permit substantial fluctuations of the conformation during the course of the MD simulation. The complete interaction energy was also calculated [27,28]. The constant pressure during MD simulation was calculated using anisotropic diagonal position scaling. The time-step used was 0.002 ps. The temperature of the system was increased gradually from 100 K to 300 K with 20 ps NPT reassemble. The target pressure was 1 atm. The Berendsen algorithm was used with a scaling factor with time constant of 0.2. The Lennard-Jones cutoff value used was 8Å. SHAKE constraints were applied to all bonds involving hydrogen atoms. Finally, 30 nsec MD simulations were run under the same conditions as the equilibration procedure. The density of the system was maintained near 1 g/cm^3^. OPLS v2007 force field was used in all calculations.

### Phylogenetic analysis

A phylogenetic tree was prepared using MEGA version 6.0 [29] employing the neighbour-joining method. For this purpose, proteins encoded by 37 RWP-RK proteins from wheat, 17 RWP-RK proteins from Brachypodium, 15 RWP-RK proteins from rice and 14 RWP-RK proteins from Arabidopsis were utilized. Initially, all the protein sequences were aligned by multiple sequence alignment (MSA) using ClustalX server 2.1 [30] and then the aligned file was uploaded in MEGA version 6.0 to generate a phylogenetic tree. Bootstrap values for the phylogenetic tree were calculated as percentage of 1000 iterations. The evolutionary distances (expressed as number of aa differences per site) were computed using the method suggested by Nei and Kumar [31].

### qRT-PCR analysis

#### Plant growing conditions

Seeds of contrasting wheat genotypes, namely C306 (low NUE) and HUW468 (high NUE), were surface sterilized with 0.1% HgCl_**2**_ for 2 min followed by 5–6 washings with distilled water. The seedlings were raised in hydroponic solution under controlled conditions at National Phytotron Facility, ICAR-IARI, New Delhi following the method described earlier [32]. Five days after germination, the seedlings were transferred to plastic containers (10 L capacity) in Hoagland solution with low (10 μM) and optimum (7.5 mM) N concentrations. The nutrient solution was changed on every third day in each treatment throughout the experiment. Fresh leaf samples for DNA were collected from 21, 24 and 25 days old seedlings of the control (optimum N) treatment. Samples were also collected from seedlings grown in low N on 21 days. In another set, after 21 days of growth at low N, the plants were completely starved of N (N starvation) and were grown for next three days with no supply of N. Root and shoot samples were collected on third day (24th day) of N starvation. Another batch of N starved seedlings was re-supplied with optimum N; root and shoot samples were then collected after 24 h (25th day). Two replications were used for each treatment in each experiment.

#### RNA isolation, cDNA synthesis, primer design and qRT-PCR analysis

Total RNA was isolated from root and shoot tissue using a TRI reagent (Sigma) followed by RNase-free DNase I (Qiagen) treatment for removal of DNA contamination. Reverse transcription reactions were performed using 2.0 **μ**g of total RNA and M-MuLV Reverse Transcriptase (Promega) according to the manufacturer’s instructions.

Primers for the four representative genes including two *NLP* and two *RKD* genes (*TaNLP2* and *TaNLP7*, *TaRKD6* and *TaRKD9*) were designed using Primer3 software (S1 Table). qRT-PCR was performed with PikoReal Real-Time PCR Systems (Thermo Scientific) using PowerUp SYBR Green Master Mix (Applied Biosystems) in three technical replicates per biological replicate. The reactions were carried out according to the following conditions: 95°C for 30 sec, 40 cycles of 95°C for 5 sec, and 60°C for 34 sec. Constitutive expression of *TaAct2* gene of wheat was used as endogenous control. The transcript abundance for each gene was normalized with the internal control, and 2^−ΔΔCt^ values (fold change) for gene expression under low N (LN), N starvation (NS) or N replete (NR) conditions vs. the control were calculated as follows: 2^−ΔΔCt^ = [(^Ct^LN/NS/NR test − ^Ct^LN/NS/NR *TaAct*) − (^Ct^cont test − ^Ct^cont *TaAct*)] [33]. A negative control was also incorporated for each primer pair.

#### Statistical analysis of qRT-PCR results

The results of qRT-PCR for expression of each of the four genes separately in roots and shoots were subjected to analysis of variances (ANOVA); for this purpose the means of two replications were used, since replications did not differ. Significance of variances over time (different durations) and space (root and shoot) and also the effect of genetic background (two cultivars) were tested for significance.

## Results and discussion

### Identification, chromosomal assignment and structure of *TaRKD* and *TaNLP* genes

In wheat, we identified 37 *RWP-RK* genes that were orthologous to only 13 of the 18 Brachypodium genes that were used as queries. The wheat genes corresponding to the remaining five Brachypodium genes must have been lost during evolution or may be available in wheat genotypes, other than Chinese Spring (CS) for which whole genome sequence was used in the present study (Table 1).

**Table 1:** A list of *RKD* and *NLP* genes of Brachypodium and their putative orthologs in wheat.

Brachypodium		Wheat			
Gene name	Gene id	Gene name	Gene id	Coordinates	Splice* variants
*BdRKD1*	Bradi2g40920	*TaRKD1-7A*	TraesCS7A01G135900	88824822-88827991	-
*BdRKD3*	Bradi1g45170	*TaRKD3-7A*	TraesCS7A01G188500	144,609,188-144,611,233	-
		*TaRKD3-7B*	TraesCS7B01G093300.3	106945174-106949546	3
		*TaRKD3-7D*	TraesCS7D01G189600	142,617,139-142,619,184	-
*BdRKD4*	Bradi3g59410	*TaRKD4-6A*	TraesCS6A01G307500	541595645-541596814	-
		*TaRKD4-6B*	TraesCS6B01G336100	591264113-591265265	-
		*TaRKD4-6D*	TraesCS6D01G286800	395390576-395391730	-
*BdRKD6*	Bradi5g18430	*TaRKD6a-2A*	TraesCS2A01G404500	659903623-659906083	-
		*TaRKD6a-2B*	TraesCS2B01G422400	607548491-607552287	-
		*TaRKD6a-2D*	TraesCS2D01G401400.2	516356306-516358927	3
		*TaRKD6b-2A*	TraesCS2A01G404400	659890344-659895450	-
		*TaRKD6b-2B*	TraesCS2B01G422300	607535903-607541394	-
		*TaRKD6b-2D*	TraesCS2D01G401300	516326169-516331225	-
*BdRKD9*	Bradi2g08600	*TaRKD9-3A*	TraesCS3A01G167700	174283498-174288783	-
		*TaRKD9-3B*	TraesCS3B01G198700	226085095-226090862	-
		*TaRKD9-3D*	TraesCS3D01G173800	155555673-155560729	-
*BdRKD10*	Bradi1g45165	*TaRKD10-7A*	TraesCS7A01G188400	144606378-144607441	-
		*TaRKD10-7D*	TraesCS7D01G189500	142612636-142613723	-
*BdRKD11*	Bradi4g39766	*TaRKD11-7A*	TraesCS7A01G497400	688153529-688155092	-
*BdNLP1*	Bradi1g76340	*TaNLP1-5A*	TraesCS5A01G497800	664809410-664812925	-
		*TaNLP1-4B*	TraesCS4B01G325500.1	616306017-616309581	2
		*TaNLP1-4D*	TraesCS4D01G322400	484402330-484406232	-
*BdNLP2*	Bradi4g37147	*TaNLP2-5A*	TraesCS5A01G349500	552522546-552526933	-
		*TaNLP2-5B*	TraesCS5B01G350900	531537886-531542381	-
		*TaNLP2-5D*	TraesCS5D01G355900.1	437341555-437346062	3
*BdNLP3*	Bradi4g20720	*TaNLP3-4A*	TraesCS4A01G230700	539755046-539761346	-
		*TaNLP3-4B*	TraesCS4B01G085200	85695720-85702042	-
		*TaNLP3-4D*	TraesCS4D01G083200	57070141-57075289	-
*BdNLP4*	Bradi5g23300	*TaNLP4-2A*	TraesCS2A01G352400.2	592852144-592856156	2
		*TaNLP4-2B*	TraesCS2B01G370400.1	528202422-528206446	2
		*TaNLP4-2D*	TraesCS2D01G350400	448208294-448212302	-
*BdNLP5*	Bradi3g03170	*TaNLP5-6A*	TraesCS6A01G102400.2	71451366-71454100	2
		*TaNLP5-6B*	TraesCS6B01G130800.1	127326253-127328935	2
		*TaNLP5-6D*	TraesCS6D01G091000	56850274-56852939	-
*BdNLP7*	*Bradi2g08177*	*TaNLP7-3A*	TraesCS3A01G159600	159422818-159428889	-
		*TaNLP7-3B*	TraesCS3B01G190300	202924394-202930308	-
		*TaNLP7-3D*	TraesCS3D01G166900	141206188-141212054	3

*A dash (-) in this column means occurrence of a single transcript (no splice variants)

Alternate splicing, which is of common occurrence in plant cells [34] was also examined during the present study involving RWP-RK genes; 2–3 splice variants were available in nine of the 37 genes, the remaining 28 genes each producing a single transcript. Of the nine genes with splice variants, two *TaRKD* genes and two *TaNLP* genes each gave three splice variants, while five *TaNLP* genes produced two splice variants each (Table 1). The occurrence of splice variants was not a surprise, because according to some estimates, >60% of intron-containing genes in plants undergo alternative splicing (AS) producing splice variants. AS has been shown to play an important role in plant growth, development, and responses to external cues [34–36].

#### Triplicate homoeologues in wheat

**Fig 1:**
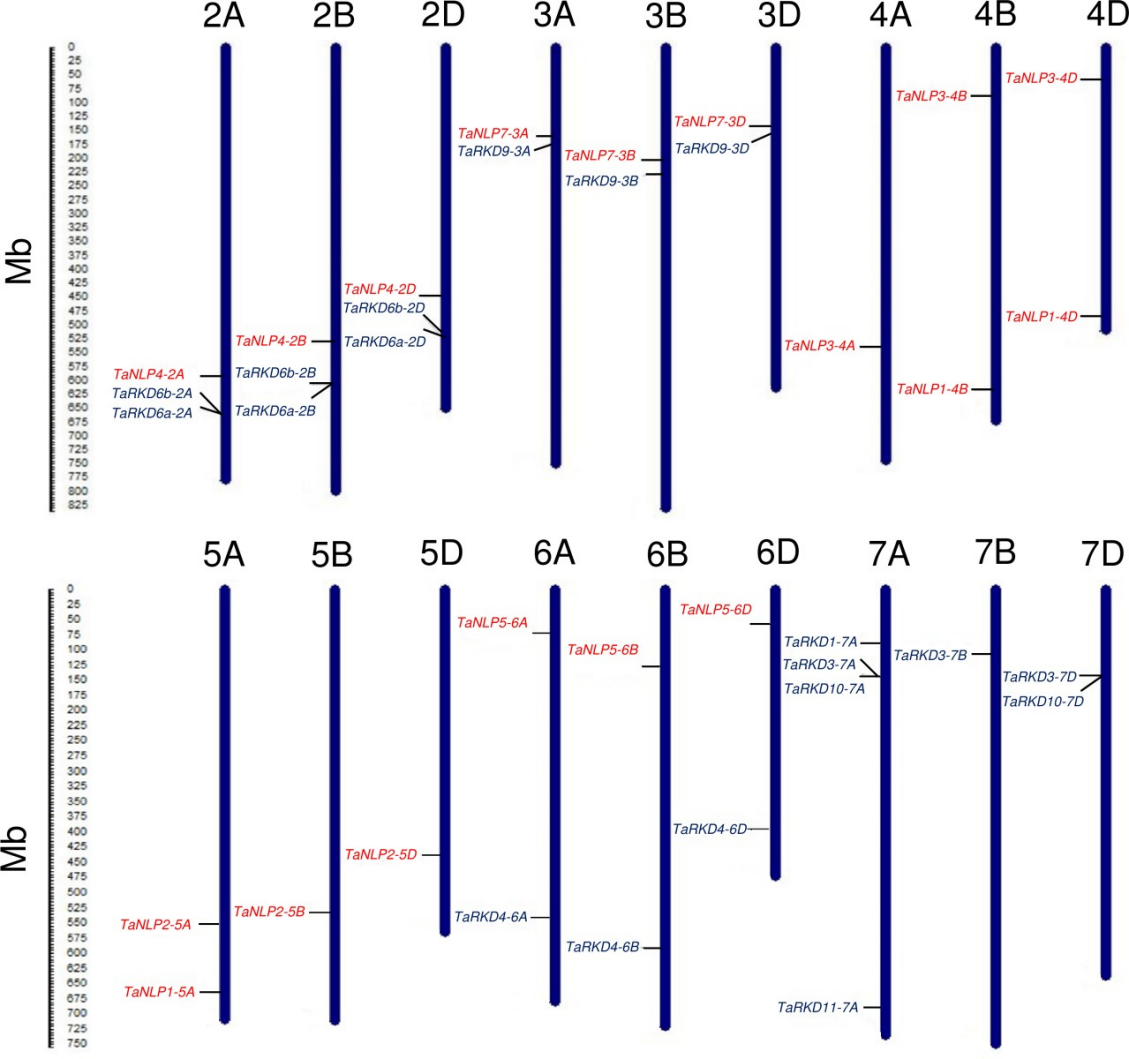
Distribution of 37 wheat *TaRWP-RK* genes (19 *TaRKD* and 18 *TaNLP*) on 18 chromosomes belonging to six homoeologous groups. Chromosomes are represented by blue solid vertical bars. *TaRKD* genes are written in black and *TaNLP* genes are written in red colour.

The 19 *TaRKD* genes (with duplicate genes on 2A, 2B and 2D) were orthologs of 7 *BdRKD* genes, and were distributed on 12 wheat chromosomes belonging to four homoeologous groups [2, 3, 6 and 7; 7A carried four genes and 2A, 2B, 2D (each as tandem repeats) and 7D carried two genes each]. Similarly, the 18 *TaNLP* genes (orthologs of 6 *BdNLP* genes) belonged to only five of the seven homoeologous groups, there being no gene on homoeologous groups 1 and 7, and there being two genes each on 4B, 4D and 5A (Table 1 and and Fig 1). The wheat genes and Brachypodium genes were largely present on corresponding homoeologous chromosomes, even though the number of chromosomes in Brachypodium is *n* = *x* = 5 (analogous to maize with *n* = 2*x* = 10) as against x = 7 in wheat. Three homoeologous groups (2, 3 and 6) carried both *RKD* and *NLP* genes of wheat; homoeologous groups 4 and 5 carried only *TaNLP* genes, while homoeologous group 7 carried only *TaRKD* genes; the homoeologous group 1 carried no *RWP-RK* genes.

If we assume that we identified all available wheat *TaRWP-RK* genes during our study, we should have obtained 54 wheat genes against 18 Brachypodium genes; even against 13 Brachypodium genes, which were found to have RWP-RK orthologs, 39 genes were expected but we were able to identify only 37 wheat genes (along with tandem duplicates), because not more than two genes were available for each of the three Brachypodium genes (*BdRKD1*, *BdRKD10* and *BdRKD11*). The missing genes in wheat included many more *RKD* genes (corresponding to 7 Brachypodium *RKD* genes), and relatively fewer *NLP* genes (corresponding to one Brachypodium gene *BdNLP6*). These missing genes must have been lost or diverged significantly during evolution of wheat. The data also suggest that the *NLP* genes are relatively more conserved than *RKD* genes. As mentioned earlier, *NLPs* play an important role in the regulation of genes involved in nitrate assimilation and other metabolic/regulatory processes associated with NUE, while *RKDs* play an important role in gametogenesis and embryogenesis [4–10,37]. Additional functions and networks involving these *RKD* and *NLP* genes may be discovered in future.

It is now well established that Brachypodium chromosomes 1, 2 and 3, each belongs to more than one homoeologous groups of wheat, while chromosomes 4 and 5 correspond to homoeologous groups 5 and 2, respectively. Since wheat genes are largely triplicate in nature, the three genes corresponding to an individual Brachypodium gene should be located on three wheat chromosomes of the same homoeologous group. There were following exceptions to this expectation: (*i*) *TaRKD1-7A*, which corresponds to *BdRKD1* located on Brachypodium chromosome 2 (homoeologous groups 1 and 3), and (*ii*) four genes [*TaRKD11-7A*, which corresponds to *BdRKD11* and *TaNLP3-4A*, *TaNLP3-4B* and *TaNLP3-4D*, which correspond to *BdNLP3*] correspond to Brachypodium chromosome 4 (wheat homoeologous group 5) (see Table 1). A suitable explanation for this discrepancy is not available at present, although this may turn out to be due to possible cryptic translocations involving non-homoeologous wheat chromosomes of groups 1, 3, 4, 5 and 7. Such a situation has been reported while studying the collinearity of the *Sh2*/*A1* orthologous region in rice, sorghum, maize and species of Triticeae [38]. The *Sh2*, *X1*, *X2* and *A1* genes are located on rice chromosome 1 and maize chromosome 3, which are homoeologous to group 3 chromosomes of wheat. Although the genes *X2* and *A1* have maintained a syntenic position on homoeologous chromosomes in wheat, maize, sorghum and rice, the other two genes (*Sh2* and *X1*) are mapped on chromosome 1A of *T*. *monococcum* and *Ae*. *tauschii*, 1H of barley and group 1 chromosomes of wheat. The above transfer of *Sh2* and *X1* genes onto the non-homoeologous group 1 chromosomes in Triticeae species has been attributed to translocation or transposition [38]. In fact, frequent micro-rearrangements between Triticeae and rice have been documented [39] suggesting a need for caution in comparative genomics studies involving model plant systems such as Arabidopsis, rice and Brachypodium.

There are also examples, where three wheat genes corresponding to the same Brachypodium gene belong to more than one homoeologous groups. For instance, the three genes that correspond to gene *BdNLP1* belong to homoeologous groups 4 (*TaNLP1-4B* and *TaNLP1-4D*) and 5 (*TaNLP1-5A*). This is not unexpected, because occurrence of a historical translocation between chromosome 4A and 5A has been reported [40], which was also confirmed recently through sequence-based analysis [41]. Known homoeology of Brachypodium chromosome 1 carrying *BdNLP1* to four wheat homoeologous groups (2, 4, 5 and 7) also explains this anomaly. We also investigated the occurrence of possible tandem and segmental duplication of wheat *RWP-RK* genes. A comprehensive gene duplication analysis showed that one pair of *TaRKD* genes each were arranged in tandem duplication on group two chromosomes *i*.*e*. chromosome 2A (*TaRKD6a-2A* and *TaRKD6b-2A*), chromosome 2B (*TaRKD6a-2B* and *TaRKD6b-2B*) and chromosome 2D (*TaRKD6a-2D* and *TaRKD6b-2D*) (Fig 1). Similar tandem duplication events have also been reported in mitogen activated protein kinase kinase kinase (MAPKKK) gene family in wheat. This kind of duplication events are known to contribute towards the expansion of gene families in wheat and other species [42].

#### Gene lengths

The length of individual *TaRKD* genes ranged from 1064 to 5768 bp, while that of *TaNLP* genes ranged from 2865 to 7340 bp, suggesting that *TaNLP* genes are little longer, perhaps due to the presence of an additional domain PB1. Accordingly, the coding DNA sequence (CDS) is also longer in *TaNLPs* (1680 to 2817 bp) than in *TaRKDs* (615 to 2784 bp). The lengths of wheat genes and the query genes from Brachypodium also varied, such that the length of *BdRKD* genes

(989 to 6480 bp) and *BdNLP* genes (3376 to 8010 bp) had a relatively longer range than the corresponding wheat genes. However, the range of the lengths of the CDS of the Brachypodium genes and the putative wheat genes was opposite to the range of the length of the gene sequences of the corresponding genes (Table 2). This may be attributed to the difference in size of the introns and untranslated regions (UTRs) of the genes belonging to Brachypodium and wheat. The average percent similarity of the CDS (73.84% for *RKDs* and 84.13% for *NLPs*) was higher than the average percent similarity of the gene sequences (64.18% for *RKDs* and 73.99% for *NLPs*) between wheat and Brachypodium. This suggested greater conservation of exonic sequences in the two species (Table 2). The ratio of non-synonymous and synonymous substitutions (Ka/Ks) of CDS of wheat genes with respect to CDS of the corresponding Brachypodium genes was >1 (1.094 to 1.722 for *RKD*s and 1.107 to 1.793 for *NLPs*) except for *TaRKD9*, *TaRKD10* and *TaNLP2* with Ka/Ks value of 0.120, 0.897 and 0.899, respectively. The >1 value of Ka/Ks indicates that almost all genes evolved under positive selection, which contributed towards speciation whereas *TaRKD9*, *TaRKD10* and *TaNLP2* (Ka/Ks ratio <1) genes have been subjected to purifying selection that did not alter the encoded amino acid sequence during 30–40 million years period of speciation [43].

**Table 2:** Details of CDSs and gene sequences for Brachypodium and wheat along with their percent similarity. *RWP-RK* wheat genes with targets for miRNAs are also shown.

Brachypodium			Wheat					miRNA having targets in genes with expected value
Gene name	Gene length	CDS length	Gene name	Gene length (bp)	% similarity	CDS length (bp)	% similarity	
	(bp)	(bp)						
*BdRKD1*	1501	1188	*TaRKD1-7A*	3170	60.97	1047	49.91	NA
*BdRKD3*	3727	1830	*TaRKD3-7A*	2246	84.74	2046	85.06	tae-miR9674a-5p, 2.5
			*TaRKD3-7B*	4670	74.48	2784	82.35	NA
			*TaRKD3-7D*	2700	79.2	2046	84.88	tae-miR9674a-5p, 2.5
*BdRKD4*	2665	804	*TaRKD4-6A*	1170	59.72	615	72.19	NA
			*TaRKD4-6B*	1153	60	615	72.53	NA
			*TaRKD4-6D*	1155	59.84	618	72.5	NA
*BdRKD6*	2742	1014	*TaRKD6a-2A*	2461	66.17	1038	80.51	NA
			*TaRKD6a-2B*	3797	65.4	1068	81.08	tae-miR838, 1.0
								tae-miR5049-3p, 1.5
								tae-miR1128, 2.5
								tae-miR1137a, 3.0
			*TaRKD6a-2D*	2622	65.82	1215	78.88	tae-miR838, 1.0
								tae-miR6197-5p, 2.5
			*TaRKD6b-2A*	5107	50.28	1014	80.0	NA
			*TaRKD6b-2B*	5492	49.66	1014	77.41	NA
			*TaRKD6b-2D*	5057	49.83	1014	80.32	tae-miR1120b-3p, 1.0
								tae-miR1130b-3p, 2.0
*BdRKD9*	6480	849	*TaRKD9-3A*	5286	54.17	921	82.73	NA
			*TaRKD9-3B*	5768	51.39	906	83.84	NA
			*TaRKD9-3D*	5057	54.81	909	83.87	NA
*BdRKD10*	989	384	*TaRKD10-7A*	1064	75.16	747	79	tae-miR5084, 3.0
			*TaRKD10-7D*	1088	75.16	771	79	NA
*BdRKD11*	2768	1116	*TaRKD11-7A*	1564	58.99	1110	66.83	NA
*BdNLP1*	5675	2766	*TaNLP1-5A*	4792	74.38	2712	84.77	NA
			*TaNLP1-4B*	5683	74.33	2718	83.98	NA
			*TaNLP1-4D*	3903	77.32	2700	84.62	NA
*BdNLP2*	5500	2712	*TaNLP2-5A*	5325	79.44	2700	87.21	NA
			*TaNLP2-5B*	5620	79.08	2697	87.98	NA
			*TaNLP2-5D*	5555	79.75	2697	88.2	NA
*BdNLP3*	8010	2643	*TaNLP3-4A*	6153	71.74	2697	83.68	tae-miR1118, 1.5
								tae-miR5084, 3.0
			*TaNLP3-4B*	6409	72.08	2739	84.17	tae-miR414, 1.0
								tae-miR5084, 3.0
			*TaNLP3-4D*	5149	74.01	2739	83.98	tae-miR1118, 0.5
								tae-miR414, 1.0
								tae-miR5049-3p, 3.0
								tae-miR5084, 3.0
*BdNLP4*	4258	2862	*TaNLP4-2A*	6197	73.15	2817	82.76	NA
			*TaNLP4-2B*	5562	72.24	2817	82.47	NA
			*TaNLP4-2D*	7340	73.14	2817	82.47	NA
*BdNLP5*	3376	2229	*TaNLP5-6A*	3565	70.9	2193	77.16	tae-miR5048-5p, 3.0
			*TaNLP5-6B*	2865	70.83	1680	77.29	NA
			*TaNLP5-6D*	3568	71.78	2166	77.11	NA
*BdNLP7*	5976	2829	*TaNLP7-3A*	6072	73.34	2802	88.76	NA
			*TaNLP7-3B*	6630	71.09	2802	89.01	tae-miR5085, 3.0
			*TaNLP7-3D*	5867	73.24	2802	88.76	NA

#### Distribution of exons and introns

**Fig 2:**
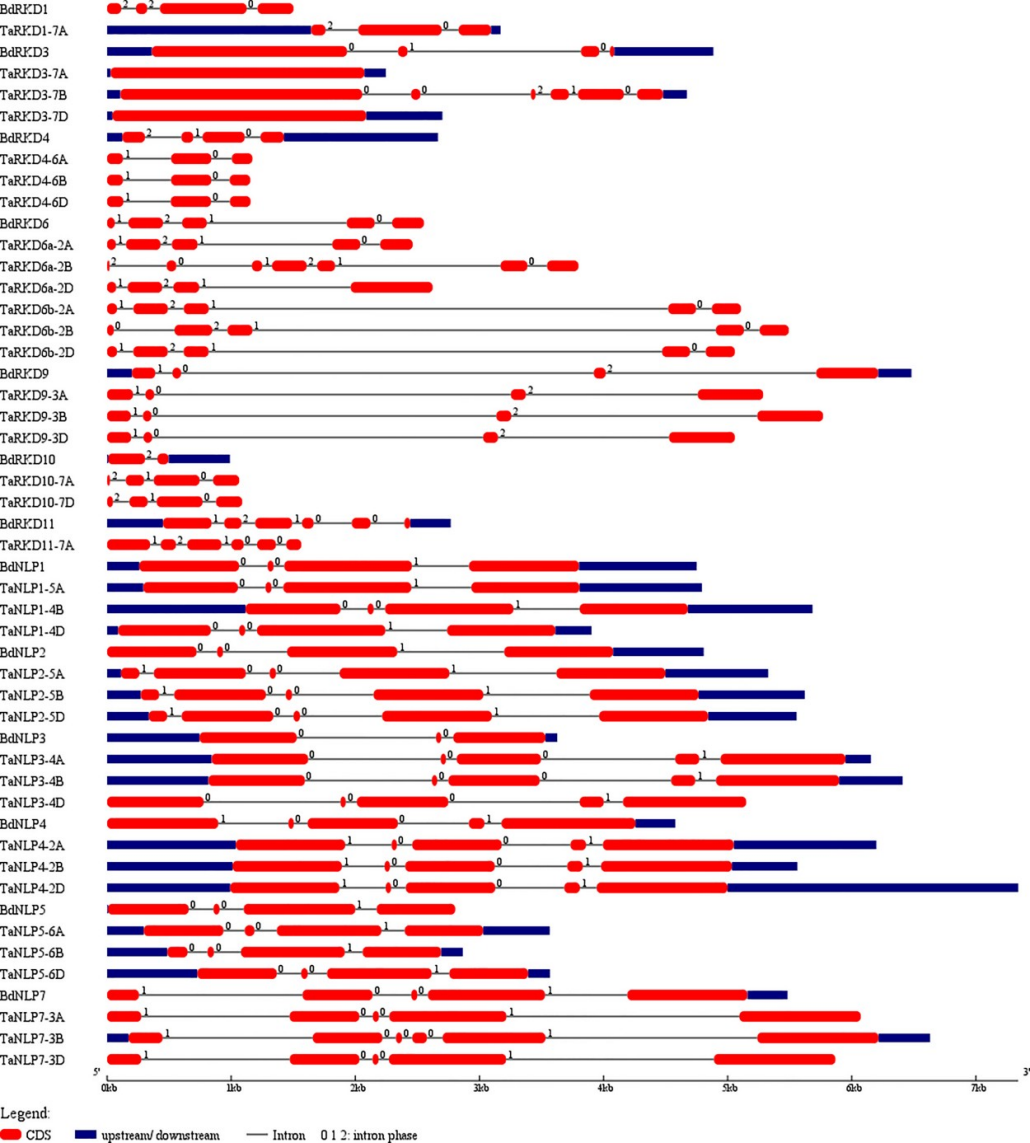
Structure of *TaRKD* and *TaNLP* genes showing distribution of exons (solid red bars), introns (black lines), upstream/downstream regions (solid blue bars) and intron phases marked as 0, 1 and 2.

The number of exons in 19 *TaRKD* genes varied from 1 to 6 (Fig 2), although the number of exons in *TaRKD* genes belonging to the same homoeologous group did not differ much (3–5 exons) except those belonging to homoeologous groups 2 and 7, which contained 1 to 6 exons. The number of exons in 18 *TaNLP* genes was generally 4 or 5. As many as 11 genes had 5 exons each and 6 genes had 4 exons each. The only exception was *TaNLP7-3B* on chromosome 3B with 6 exons (Fig 2).

The number of exons in corresponding Brachypodium genes differed; 2–6 exons were present in *BdRKD* genes and 3–5 exons were present in the *BdNLP* genes. This also suggests higher level of variation in the number of exons in genes belonging to RKD and NLP sub-families in wheat than in Brachypodium. This difference in variation of the number of exons in the genes of the two species could be attributed to deletion, addition, and merging of exons. A comparison of number of exons in the genes belonging to wheat and Brachypodium would suggest that in case of each of the two RKD genes [*TaRKD3-7A* and *TaRKD3-7D*], all the exons merged together resulting into solitary exons. Similar results showing merging of adjacent exons in *AGPase* LS gene of Arabidopsis, chickpea and potato and *AGPase* SS gene of Arabidopsis were reported by us earlier [13]. Also, an addition of an exon was noticed in *TaRKD3-7B*, so that the total number of exons in this case is 6 against 5 exons in the corresponding *BdRKD3* gene of Brachypodium. Similarly, addition of 1 to 2 exons was noticed in seven *TaNLP* genes (*TaNLP2-5s*, *TaNLP3-4s* and *TaNLP7-3B*) (Fig 2).

Intron phases were also examined in all the *TaRWP-RK* genes. In *TaRKD* genes, the intron phase 1 was more prevalent (37.93% in each) followed by intron phase 0 (36.22%) and intron phase 2 (25.86%). In *TaNLP* genes, the intron phase 0 (59.70% in each) was most frequent followed by intron phase 1 (40.29%), once again suggesting that *TaNLP* genes are more conserved than *TaRKD* genes (Fig 2). Prevalence of intron phase 1 in *TaRKD* genes suggested that the wheat genes belonging to this sub-family have undergone rapid evolution, as suggested earlier [44].

### Simple sequence repeats (SSRs) in *TaRKD* and *TaNLP* genes

As many as 24 SSRs were detected in 16 of the 37 genes, including 7 SSRs in 7 *TaRKD* genes and 17 SSRs in 9 *TaNLP* genes. Most genes carried each a single SSR except *TaNLP1-4B* and *TaNLP5-6A*, each carrying two SSRs, and *TaNLP1-4B* carrying three SSRs (S2 Table). Tri-nucleotide repeats were most frequent (14 SSRs) followed by hexa-nucleotide repeats (6 SSRs) and tetra -nucleotide repeats (4 SSRs). The abundance of tri-nucleotide repeat SSRs in the present study is in agreement with earlier reports in wheat [45]. SSRs have also been reported in genes encoding other TFs in chickpea [46] and *M*. *truncatula* [47]. In future, the polymorphism for SSRs in *TaRKD* and *TaNLP* TF genes may be examined in wheat cultivars and the polymorphic SSRs may be utilized for developing markers to be used for MAS in wheat breeding programmes for improvement of NUE in wheat.

### Micro RNA (miRNA) and their targets in *TaRKD* and *TaNLP* genes

The 37 *TaRWP-RK* genes were also examined for miRNA targets. Six of the 19 *TaRKD* genes and five of the 18 *TaNLP* genes carried targets for 13 different miRNAs; 12 of these 13 miRNAs are being reported in wheat for the first time, the only exception being tae-miR414 (see Table 2 for details). In particular, tae-miR414 targets *TaNLP3-4B* and *TaNLP3-4D*, although it is known to target six other wheat genes, which encode proteins that are either involved in metabolic (nucleosome assembly protein I and ATPase subunit 6) and developmental processes (differentially expressed in relation to the extent of cell elongation; nuclear polyadenylated RNA-binding protein NAB3) or represent TFs [TFIIA large subunit (TFIIA-L1) and other DNA-binding protein-like protein] [48]. The miR414 is also known to play important roles in some other species. For instance, it plays a role in N-metabolism in saffron (*Crocus sativus* L.) [49], drought tolerance in *P*. *patens* [50] and plant development in *Stevia rebaudiana* [51]. Another important tae-miRNA for which targets were found in *RWP-RK* genes is tae-miR838, which has been reported to have a target site in *DCL1* gene of *Medicago truncatula*, where it expresses in root nodules and thus plays a role in biological nitrogen fixation [52]. These results clearly demonstrate that besides their other functions, miRNAs are also involved in regulation of the expression of genes encoding transcription factors like RWP-RKs [53]. The present study suggests that the expression of tae-miR414 and tae-miR838 in particular may be further examined for their role in regulating the expression of *RWP-RK* genes in wheat in low-N tolerant and low N-sensitive genotypes or in a genotype responding differently under low and high N-availabilities. This will perhaps help further in manipulation of the regulatory pathways for development of wheat genotypes with improved NUE [3].

### Synteny (gene content) and collinearity (gene order)

**Fig 3:**
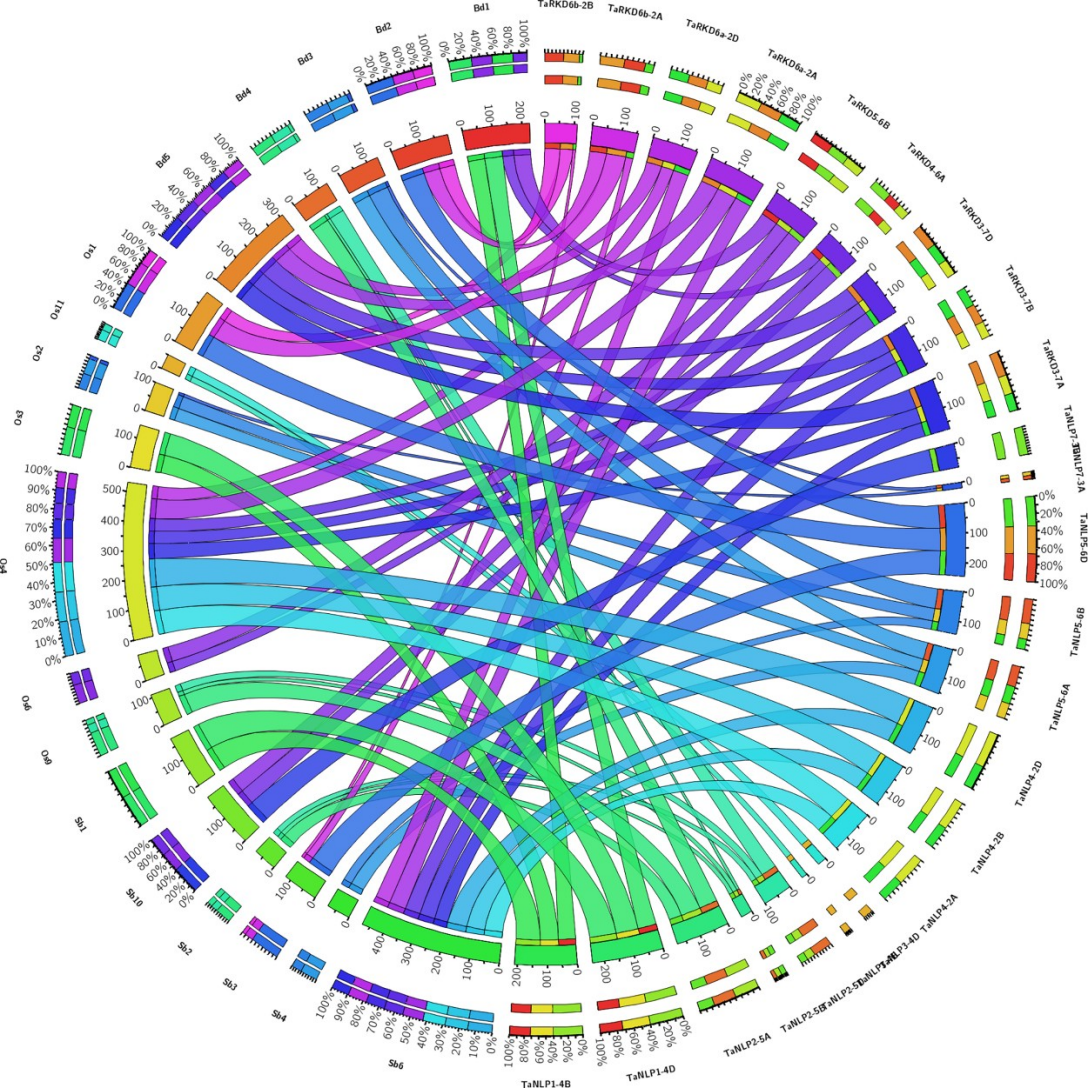
The circos visualization map showing synteny and collinearity among RWP-RK proteins of wheat, Brachypodium, rice and sorghum. The bands of different colours with variation in thickness depict different levels of synteny between wheat proteins with those of other species.

Synteny and collinearity analysis was undertaken using a block of 21 genes, including 10 genes flanking either side of each of the 19 *TaRKD* and 18 *TaNLP* genes. For this purpose, *RKD* and *NLP* genes from wheat, Brachypodium, rice and sorghum were utilized, since the data in URGI was available for only these four plant species (Fig 3). Among 37 RWP-RK genes, 9 *TaRKD* genes showed 10 to 70% synteny and 15 *TaNLP* genes showed 10 to 100% synteny with corresponding genes in three other genomes. The 9 *TaRKD* genes showed maximum average percent synteny with Brachypodium genes (53.5%), followed by sorghum genes (50.0%) and rice genes (47.5%). For 15 *TaNLP* genes, the average synteny did not differ much among three species; 9 *TaNLP* genes showed mean syntney of 58.1% with Brachypodium genes, 14 *TaNLP* genes showed mean syntney of 57.1% with rice genes and 12 *TaNLP* genes showed mean syntney of 56.8% with sorghum genes. The remaining *TaRWP-RK* genes did not show any synteny with the corresponding genes of the other three species. The collinearity within the synteny blocks for each of the *TaRKD* and *TaNLP* genes was disrupted due to insertion, deletion, duplication, and rearrangement of genes (S1 Fig). In the past, the loss of shared synteny and collinearity of some genes across grasses was attributed to gain/loss of genes and other structural changes within chromosome segments [54].

### Promoter analysis of *TaRKD* and *TaNLP* genes(Identification of cis-elements.)

Promoter analysis allowed identification of cis-acting response elements associated with different *TaRKD* and *TaNLP* genes. These cis-elements presumably allow spatial and temporal expression of these TF genes, thus indirectly regulating the expression of other genes. Most of these cis-acting response elements were conserved; only in some genes, the basic regulatory elements like TATA-box and CAAT box were absent (TATA box was absent in *TaRKD4-6D*, *TaRKD6a-2B*, *TaRKD6b-2A*, *TaRKD6b*-*2B*, *TaRKD10-7s*, *TaRKD11-7A*, *TaNLP1-5A*, *TaNLP1-4B*, *TaNLP1-4D*; CAAT box was absent in *TaRKD3-7D*, *TaRKD11-7A*, *TaNLP3-4D*) (Table 3 and S3 Table for details). However, response elements for zein regulatory metabolism and for circadian rhythm were present in *TaRKD* genes on homoeologous groups 2 (*TaRKD6a-2s*, *TaRKD6b-2A*, *TaRKD6b-2D*), chromosome 7 (*TaRKD11-7A*) and chromosomes of homoeologous group 3 (*TaRKD9-3A* and *TaRKD9-3D*); the response elements for circadian rhythm were also present in *TaNLP* genes on homoeologous group 6 (*TaNLP5-6A* and *TaNLP5-6D*).

**Table 3:** A summary of cis-acting regulatory elements for abiotic stress in promoters of *TaRKD* and *TaNLP* genes of wheat.

No.	Response elements	Response motifs and their sequences
1.	Nitrogen responsive	GCN4 (ATGA (C/G) TCAT)
2.	Biotic stress	EIRE (elicitor), TGACG (MeJa responsive), TCA (salicylic acid), Box-W1 (fungal elicitor, TTGACC), ERE (ethylene responsive)
3.	Abiotic stress	ABRE (ACGTGGC), GARE (AAACAGA), CGTCA (MeJa responsive), GC (anoxic specific, CCCCCG), ARE (anaerobic induction, TGGTTT), MBS (drought inducible, CGGTCA), motif IIb (ABA responsive, CCGCCGCGCT), TC-rich repeats (defence and stress responsive, ATTTTCTCCA), LTR (low temperature), P-box (gibberellin responsive, CCTTTTG), motif IIIb (ABA responsive), HSE (heat stress responsive, AAAAAATTTC), CCAAT box (MYB binding site, CAACGG)
4.	Light	as-2-box, AE-box, ACE, ATC (GCCAATCC), ATCC (CAATCCTC), ATLT, Box-4 (ATTAAT), Box-1, CG, chs-CMA1a (TTACTTAA),CAG (GAAAGGCAGAC), CATT (GCATTC), GA, G-Box (CACGTC/CACGAC/CACGTA/CACGTT), GAG (AGAGATG), GT-1 (GGTTAA), GATA (GATAGGG), Gap box, GATT (CTCCTGATTGGA), I-box (GATAGGG), L-box, LAMP element (CTTTATCA), MNF1 (GTGCCC(A/T)(A/T), Sp1 (CC(G/A)CCC/GGGCGG), TCCC (TCTCCCT), TCT (TCTTAC), TGG (GGTTGCCA), 3-AF1 (TAAGAGAGGAA)
5.	Tissue specific	Skn-1 (GTCAT) and GCN4 (endosperm, ATGA (C/G) TCAT), CCGTCC, CAT-box (GCCACT), Non-box (meristem), as-2-box (shoot specific), Ry element (seed specific)
6.	Circadian rhythm	CAANNNNATC
7.	O_2_ site/ Zein metabolism regulation	GATGATGTGG/GTTGACGTGA

The presence of nitrogen responsive elements (NREs) in the promoter regions of *TaRKD3-7D* and *TaNLP4-2D* suggest the role of these genes under N-starvation. One such element is GCN4 motif [ATGA (C/G) TCAT], which is the binding site for the TF GCN4, whose synthesis is regulated at the translational level. In a study in barley, GCN4 motif was shown to function as a negative regulator for hordein genes under low N condition [55].

Another 13 different response elements for abiotic stress were available in the promoters of several *TaRKD* and *TaNLP* genes. These motifs (ABRE, GARE and MBS) are known to play a role in response to heat and drought stress [56], but their role in response to N-starvation (an abiotic stress) is yet to be examined. The tissue-specific and light-responsive elements may be responsible for expression of *RKD/NLP* genes in specific tissues and during plant development [57,58]. This expression of *RKD*/*NLP* genes encoding TFs should in turn induce expression of other structural genes encoding proteins that may take part in actual metabolic processes (see next section).

### *In silico* expression of *TaRKD* and *TaNLP* genes

Spatial and temporal expression profiles of three *TaRKDs* and six *TaNLPs* (each representing three homoeologues; thus making 9 *TaRKD* and 18 *TaNLP* genes) were examined in 15 different tissues and at 10 different developmental stages. The expression was also compared under variable doses of N. For the remaining *TaRKD* genes, no *in silico* expression analysis could be performed, as no information for these genes was availbale in Genevestigator database.

#### Expression in different tissues

*In silico* expression of 27 wheat *RWP-RK* genes (three homeologos each of 3 *RKD* and 6 *NLP* genes) in 15 different wheat tissues differed (S2 Fig). Similar results were earlier reported in Arabidopsis and rice [4]. *TaRKD6*, *TaRKD9*, *TaNLP2*, and *TaNLP3* showed relatively higher but variable expression in all the 15 tissues except in few cases, where the expression was low. The results of several earlier studies are presented with our own results (Table 4). These results confirm the role of *TaRKDs* in embryogenesis and gametogenesis [59,60]. Similarly, *TaNLPs* have a role in the development of reproductive organs and roots [4,61].

**Table 4:** Results of expression analysis obtained during present study and earlier studies.

Plant species	Gene name	Expression		Reference
Wheat	*TaRKD6* and *TaRKD9*	Variable expression; highest in embryo, inflorescence, endosperm and caryopsis and moderate in crown, mesocotyl, seedling, root and coleoptile		Present study
Arabidopsis and rice	*RKDs*	Variable expression in reproductive organs		[4]
*Adiantum capillus-veneris* L	*AcRKD4*, an ortholog of *AtRKD4*	High expression in green globular bodies during somatic embryogenesis		[59]
Wheat	*TaNLP2* and *TaNLP3*	Variable expression; highest for *TaNLP2* (root tissues) and *TaNLP3* (spikelets)		Present study
Maize	*ZmNLP4* and *ZmNLP8*	High expression in roots, developing grains and other tissues		[61]

#### *In silico* expression at different developmental stages

The expression profiles of different *TaRKDs* and *TaNLPs* at 10 different development stages in wheat showed their variable expression (S3 Fig). Following three genes had relatively higher expression at some specific developmental stages: (*i*) *TaNLP2* at booting, tillering and stem elongation stages, (*ii*) *TaRKD9* at booting and germination stages, and (iii) *TaRKD6* at the time of booting, germination, ripening, dough and milk developmental stages. Similar results were also reported for *ZmNLP2* in maize [61]. Taken together, these results suggested developmental and tissue specific expression of *TaRKDs* and *TaNLPs* at different developmental stages.

#### *In silico* expression under variable N inputs

The expression of different *TaRKD* and *TaNLP* genes was examined under variable doses of N using data available in Genevestigator. None of the *TaRKD* genes showed significant differential expression (fold change ≥2 and p value 0.05) under variable doses of N supply. However, in *Chlamydomonas*, *MID* gene containing RWP-RK motif (a characteristic of the wheat *RKD* genes) was shown to play a role in gamete formation under N starvation [8]. Similar studies need to be conducted in wheat and other eukaryotes also [4].

**Fig 4:**
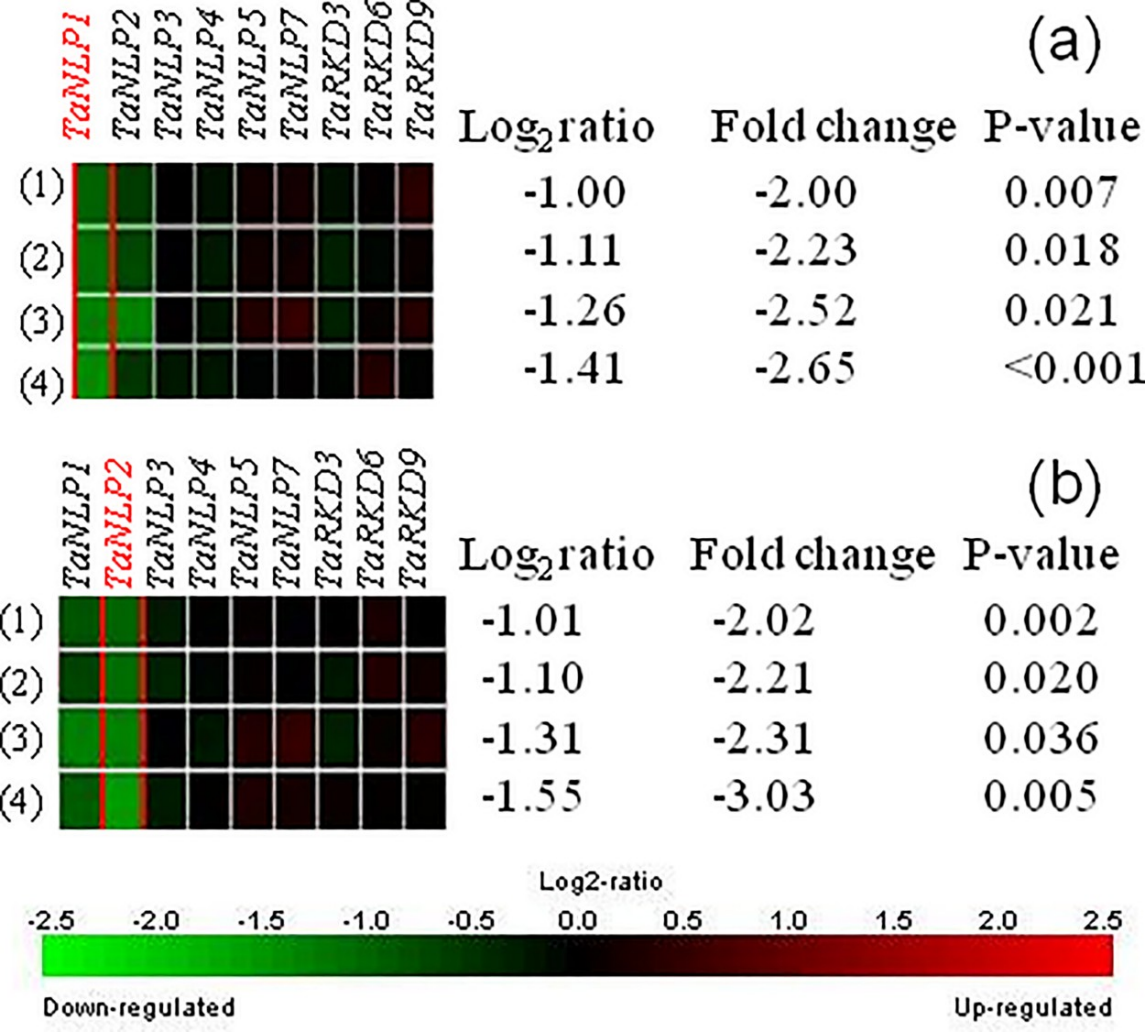
Results of *in silico* expression analysis of *TaRKD* and *TaNLP* genes of wheat under varying doses of nitrogen in four different experiments. (a) Experiment (1)-(3) showing expression at 21 daa under 200 kg N/ha vs. 50 kg N/ha (used as control),using genotype Istabraq in 1^st^, Hereward in 2^nd^ and Soissons in 3^rd^, and experiment (4) showing expression at 7 daa under192 kg N/ha vs. 48 kg N/ha (used as control), and (b) Experiment (1)-(2) showing expression at 7 daa at 200 kg N/ha vs. 50 kg N/ha (used as control) and experiment (3)-(4) showing expression at 21 daa under 200 kgN/ha vs. 50 kg N/ha (used as control) using genotype Riband in 1^st^ and 3^rd^ and Soissons in 2^nd^ and 4^th^. Genes showing significant differential expression are indicated with red colour.

In contrast to *TaRKDs*, two *TaNLPs* (*TaNLP1*, and *TaNLP2*) exhibited up-regulation at low doses of N (50 kg/ha) relative to that at higher doses of N (200 kg N/ha; Fig 4A and 4B). These results are in agreement with those reported in an earlier study in Arabidopsis, where *AtNLP5*, *AtNLP8* and *AtNLP9* showed relatively higher expression during N-starvation [4]. Thus, it appears that the low doses of N induce higher expression, while high doses inhibit expression of *NLP* genes. However, these *in silico* results need to be validated in wet-lab studies in wheat.

In contrast to our own results, where no other genes except *TaNLP1* and *TaNLP2* showed differential expression, in several other studies, *NLP7* was reported to be the most important *NLP* gene. For instance, under condition of low dose of nitrate, *AtNLP7* induced differential expression of a large number of genes including nitrate transporter (*NRT2*.*1*), nitrate reductase (*NIA1*), nitrite reductase (NiR) and glutamine synthetase 2 (GS) [10,62]. Also, *nlp7* mutants (but no other *NLP* mutant) in Arabidopsis exhibited abnormal phenotype (rosette structure, delayed growth and flowering) under N starvation. We also identified 13 wheat orthologs of a number of downstream Arabidopsis genes that are differentially expressed; only four downstream genes (*4CL1*, *CHX17*, *SIR* and *AMP* dependent synthetase) showed significant differential expression under variable doses of nitrogen (data not presented). Since, *TaNLP7* did not show significant variation in expression in the wheat microarray data set used by us during the present study, the role of TaNLP7 TF in regulation of the expression of a number of downstream wheat genes needs to be further examined.

### Characterization of proteins

The lengths of predicted proteins for 19 *TaRKD* genes were more variable (204 aa for *TaRKD4-6B* to 927 aa for *TaRKD3-7B*) than the predicted proteins for the 18 *TaNLP* genes (559 aa for *TaNLP5-6B* to 938 aa for each of the three *TaNLP4* genes on group 2 chromosomes) (Table 5). The same pattern is available in BdRKD (127aa to 609 aa) and BdNLP proteins (742 aa to 953 aa). Also, relative to TaRKDs (52.99 to 81.73, mean 66.74), TaNLPs had higher similarity (73.30 to 89.78; mean 81.31) with corresponding Brachypodium proteins, once again suggesting higher level of conservation of NLPs (also inferred above at the gene level).

**Table 5:** Details of Brachypodium and wheat RWP-RK proteins sequences and their conserved domains (with per cent similarity of wheat proteins with Brachypodium proteins).

Brachypodium proteins				Wheat proteins				
Protein name	Protein length (aa)	RKD domain position	PB1 domain position	Protein name	Protein length (aa)	% similarity	RKD domain position	PB1 domain position
		(size in aa)	(size in aa)				(size in aa)	(size in aa)
BdRKD1	385	244–292	-	TaRKD1-7A	348	52.99	206–254 (49)	-
BdRKD3	609	-	-	TaRKD3-7A	681	8216	-	-
				TaRKD3-7B	927	77.46	803–850 (48)	-
				TaRKD3-7D	681	81.44	-	-
BdRKD4	267	150–197	-	TaRKD4-6A	204	58.08	97–142 (46)	-
				TaRKD4-6B	204	59.09	95–142 (48)	-
				TaRKD4-6D	205	57.07	96–143 (48)	-
BdRKD6	337	197–245	-	TaRKD6a-2A	345	68.47	203–251 (49)	-
				TaRKD6a-2B	355	70.45	216–264 (49)	-
				TaRKD6a-2D	404	68.25	204–252 (49)	-
				TaRKD6b-2A	337	69.63	204–252 (49)	-
				TaRKD6b-2B	337	68.10	204–252 (49)	-
				TaRKD6b-2D	337	70.55	204–252 (49)	-
BdRKD9	282	41–88	-	TaRKD9-3A	306	81.07	48–95 (48)	-
				TaRKD9-3B	301	81.88	42–89 (48)	-
				TaRKD9-3D	302	82.25	43–90 (48)	-
BdRKD10	127	43–90	-	TaRKD10-7A	248	70.53	124–171 (48)	-
				TaRKD10-7D	256	71.43	132–179 (48)	-
BdRKD11	371	312–360	-	TaRKD11-7A	369	53.98	286–334 (49)	-
BdNLP1	921	604–652	825–905	TaNLP1-5A	903	82.62	591–639 (49)	812–892 (81)
				TaNLP1-4B	905	81.66	593–641 (49)	814–894 (81)
				TaNLP1-4D	899	82.77	587–635 (49)	808–888 (81)
BdNLP2	903	589–637	806–886	TaNLP2-5A	899	83	585–633 (49)	802–882 (81)
				TaNLP2-5B	898	85.12	584–632 (49)	801–881 (81)
				TaNLP2-5D	898	85.12	584–632 (49)	801–882 (81)
BdNLP3	880	540–588	783–863	TaNLP3-4A	898	76.37	541–589 (49)	799–877 (79)
				TaNLP3-4B	912	76.09	560–608 (49)	817-895(79)
				TaNLP3-4D	912	75.63	560–608 (49)	817–895 (79)
BdNLP4	953	572–620	854–934	TaNLP4-2A	938	81.08	565–613 (49)	837–917 (81)
				TaNLP4-2B	938	80.97	565–613 (49)	837–917 (81)
				TaNLP4-2D	938	80.75	565–613 (49)	837–917 (81)
BdNLP5	742	511–558	636–729	TaNLP5-6A	730	72.29	499–546 (48)	625–695 (71)
				TaNLP5-6B	559	74.23	328–375 (48)	454–546 (93)
				TaNLP5-6D	721	73.39	489–536 (48)	615–707 (93)
BdNLP7	942	598–646	849–929	TaNLP7-3A	933	89.78	586–634 (49)	840–920 (81)
				TaNLP7-3B	933	89.68	586–634 (49)	840–920 (81)
				TaNLP7-3D	933	89.89	586–634 (49)	840–920 (81)

Note: A dash (-) means absence of domain

#### Functional domains

More information is available for RKD domain (46-49aa) in both RKD and NLP proteins relative to NLP’s PB1 domain (71-93aa) that is separated from RKD domain by 127-273aa (Table 5). The RKD domain was present in 17 of the 19 TaRKDs (except TaRKD3-7A and TaRKD3-7D) and occurred towards the C-terminus except in those encoded by genes on homoeologous group 3 chromosomes, where the RKD domain was available towards the N-terminus. The RKD domain contains a consensus sequence RWPXRK that has been described as a characteristic feature of RWP-RK family of TFs in plants [4]. However, N-terminal RKD domain in two TaRKD proteins, namely TaRKD3-7A and TaRKD3-7D, is deficient for more than half of the downstream region with RWPXRK, which is responsible for DNA-binding function. A similar situation was reported in OsNLP6 [4]. One may speculate that these proteins (deficient for more than half of RKD domain) must have lost their DNA binding properties and must have either acquired new function or become non-functional [4]. Regarding PB1 domain, its role in interaction of NLPs with other proteins has been demonstrated in Arabidopsis, but no such information is available for wheat NLPs.

#### Physicochemical properties of proteins

The molecular weight of predicted TaRKD proteins varied from 23.75 to 103.69 KD and that of TaNLP proteins ranged from 98.56.26 to 102.31 KD. The isoelectric point (pI) of all TaRKD proteins was within the alkaline range except in case of TaRKD10, TaRKD11, TaRKD3-7B and TaRKD3-7D proteins, where the pI was within the acidic range. However, the situation of the TaNLP proteins differed, where the pI ranged from high (alkaline range) to low (acidic range) [63]. All TaRKD and TaNLP proteins had unstable nature, making it difficult to obtain these proteins in pure crystalline state for a study of their crystalline structure [64]. However, the higher aliphatic index for the TaRKD proteins (range: 71.63–86.10) relative to that of TaNLP proteins (range: 71.02–79.28), suggests relatively higher stability of the TaRKDs at wider range of temperatures [65]. Grand Average of Hydropathy (GRAVY) ranged from -0.153 to -0.629 for TaRKD proteins and from -0.319 to -0.469 for TaNLP proteins suggesting hydrophilic nature of these proteins (S4 Table). The physicochemical properties of the RWP-RK proteins from other plant species are not reported and hence the results of the present study could not be discussed.

#### 3D structure

**Fig 5:**
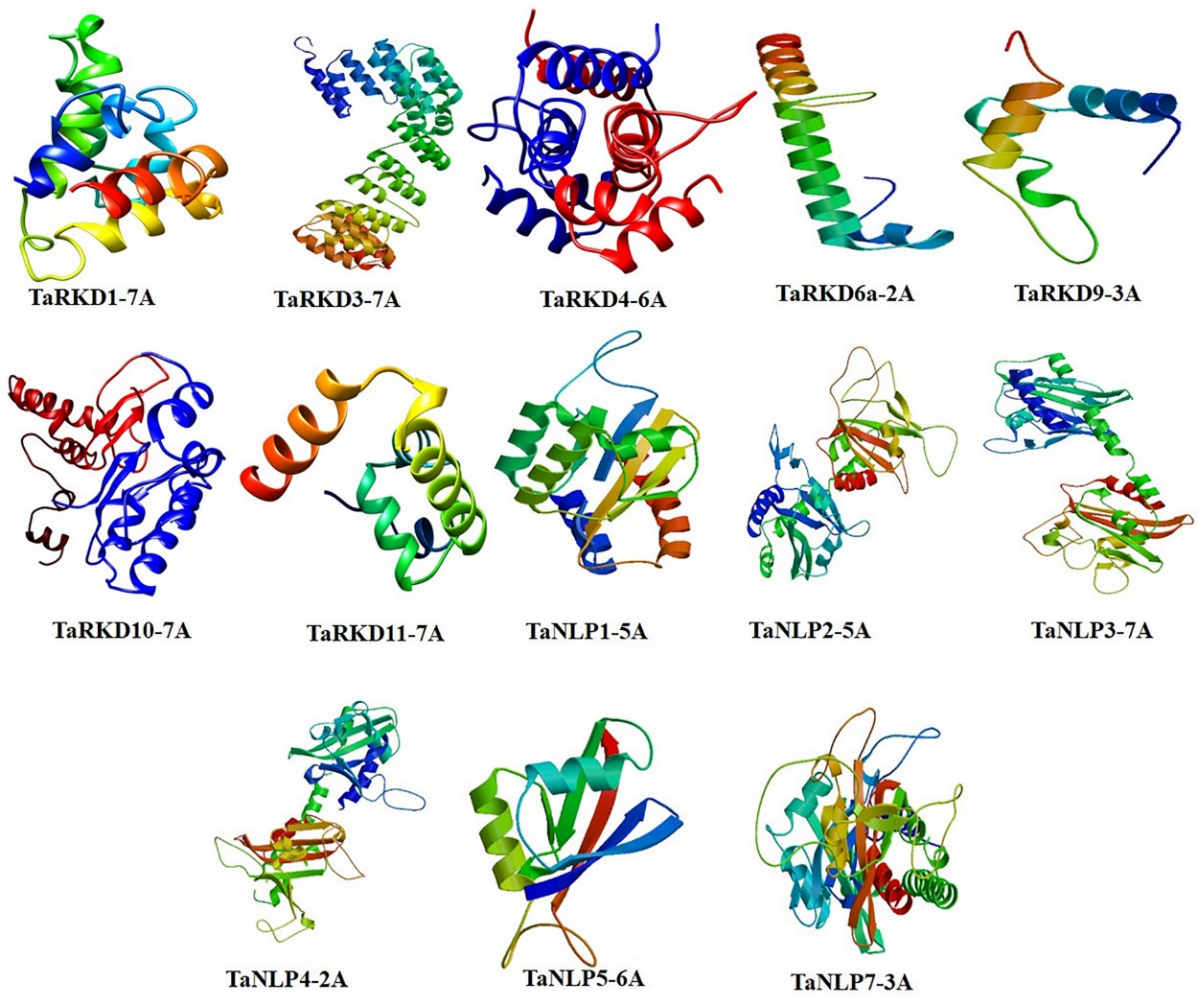
3D structures of TaRKD and TaNLP proteins. In all figures, spirals are helices, broad strips with arrow-head are β-pleated sheets and thin loops are coils.

*In silico* 3D structures were determined for eight TaRKD and six TaNLP representative proteins (Fig 5), which shared 50–80% similarity with the corresponding Brachypodium structures used as template. This level of structural similarity was adequate for analysis of 3D protein structures (a minimum of 30% similarity is needed) that were determined using Swiss-Model algorithm (S5 Table). Ramachandran plots indicated a high proportion of amino acids in the favoured region (i.e. 78.3% in TaRKD9-3A to 96.3% in TaRKD6-2A; 75.9% in TaNLP5-6A to 84.7% in TaNLP3-4A) suggesting satisfactory geometry of the predicted 3D structures (S5 Table). The use of Ramachandran plot for evaluation of the accuracy of protein structures is known and has been emphasized in several recent studies [27, 66]. The 3D structures of TaRKDs and TaNLPs have been submitted to Protein Model Database (PMDB) (https://bioinformatics.cineca.it/PMDB/) [67], which can be freely accessed (S5 Table).

#### Alignment, subcellular localization and functional annotation of 3D structures

The 3D structures of three TaRKDs and four TaNLPs had significant per cent similarity with corresponding 3D structures of BdRKDs [range of similarity = 36.97% (TaRKD3-7A) to 88.14% (TaRKD6-2A)] and BdNLPs [range of similarity = 75.77% (TaNLP4-2A) to 95.99 (TaNLP7-3A)], respectively (S4 Fig and S6 Table). The 3D structures of the remaining four proteins from RKD sub-family (TaRKD1-7A, TaRKD4-6A, TaRKD9-3A, TaRKD10-7A) and two proteins from NLP sub-family (TaNLP1-4B and TaNLP5-6A), however, did not have significant similarity (<30%) with 3D structures of the respective Brachypodium proteins. This may be attributed to the possible divergence of the amino acid sequences of the corresponding wheat and Brachypodium proteins.

All TaRKDs and TaNLPs (except TaRKD3-7s and TaRKD6b-2B) were localized in the nucleus providing support for their function as transcription factors (S7 Table) [27]. The functional annotation analysis also suggested their involvement in different activities including DNA binding, metal ion binding, protein binding and ATP binding, thus influencing several biological processes including cellular, metabolic and biosynthetic processes (S5 Fig). In particular, NLPs should respond to nitrate signals, through binding to cis-elements of relevant genes [6].

### Molecular docking and molecular dynamics simulations analysis

**Fig 6:**
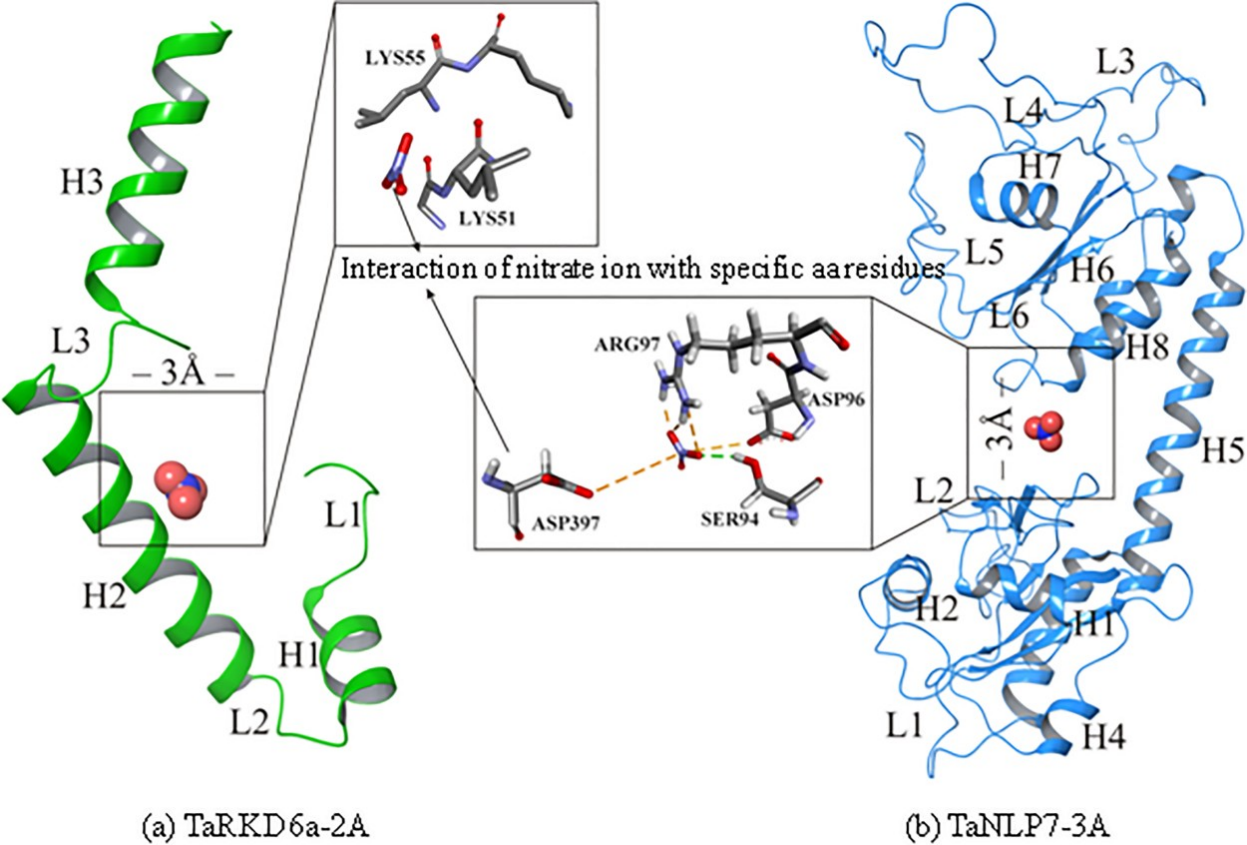
Interaction of nitrate ion with specific amino acid residues of (a) TaRKD6-2A and (b) TaNLP3-7A proteins.

Since, the protein structures of the two TFs (TaRKD6-2A and TaNLP7-3A) showed maximum amino acids in the favoured regions in Ramachandran plots (S5 Table), we used these two representative protein structures, one from each sub-family, for molecular docking and molecular simulation analysis. The active region of TaRKD6-2A was situated between helix H2 and loop L3. Amino acid residues from THR46 –LYS57 of H2 and GLY62 –ARG65 of loop3 were involved in the formation of this active region. Nitrate ion that functions as a ligand did not show any hydrogen bond formation but showed good contact with LYS51 and LYS55 within 3 Å in active regions (Fig 6A). The active site of TaNLP7-3A covered helix H8 and loops L2 and L6. Nitrate ion formed one hydrogen bond (2.04 Å) with SER94 and four salt bridge interactions involving the following three amino acid residues: ASP397 (5.40Å), ARG97 (2.05Å & 3.03Å) and ASP96 (3.93Å) (Fig 6B). The docking scores of TaRKD6-2A (4.59) and TaNLP7-3A (5.37) showed compact binding affinity with pocket fitting in the active regions suggesting stable conformation of both the protein-nitrate ion complexes.

**Fig 7:**
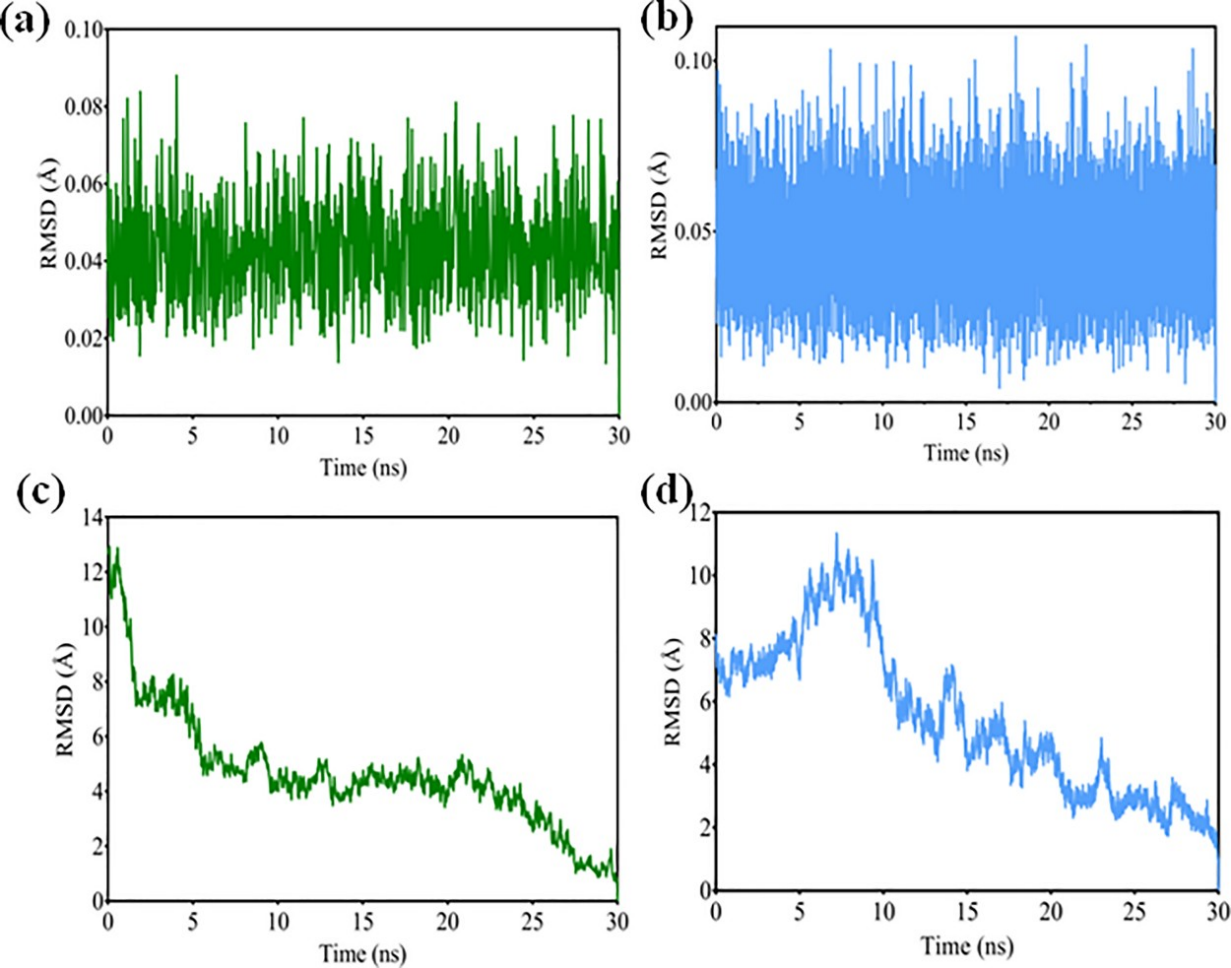
RMSD plots of nitrate ion during binding with TaRKD6-2A (a and c) and TaNLP3-7A (b and d) obtained using Prizm software.

The effect of nitrate ion on the binding position of both the above protein structures (TaRKD6-2A and TaNLP7-3A) showed stability values in the acceptable range of <3.0 Å (0.02Å – 0.07Å for TaRKD6-2A and 0.03Å – 0.08Å for TaNLP7-3A). RMSD values suggested that nitrate ion remained bound in the binding pocket and stabilized both the protein structures during simulation analysis (Fig 7A and 7B). The RMSD values of complex involving TaRKD6-2A protein showed fluctuations during 1 nsec to 13 nsec trajectory between 4.2Å – 8.5Å, after which RMSD values got stabilized and converged at 2.1Å – 2.4Å distance in fixed range. In the complex involving TaNLP7-3A, protein backbone atoms showed higher RMSD values relative to TaRKD6-2A during 1 nsec to 19 nsec at 4.05Å - 6.88Å distance. This may be due to the availability of more loop regions in the 3D structure of TaNLP7-3A. These loop regions may be responsible for higher fluctuations in the 3D structure. After 19 nsec, RMSD values decreased and were stabilized at 1.25Å – 2.82Å distance (Fig 7C and 7D). The RMSD values obtained during 0 nsec to 1 nsec trajectory in the initial stages of simulation analysis were not considered due to large thermal changes.

### Phylogenetic analysis of proteins

**Fig 8:**
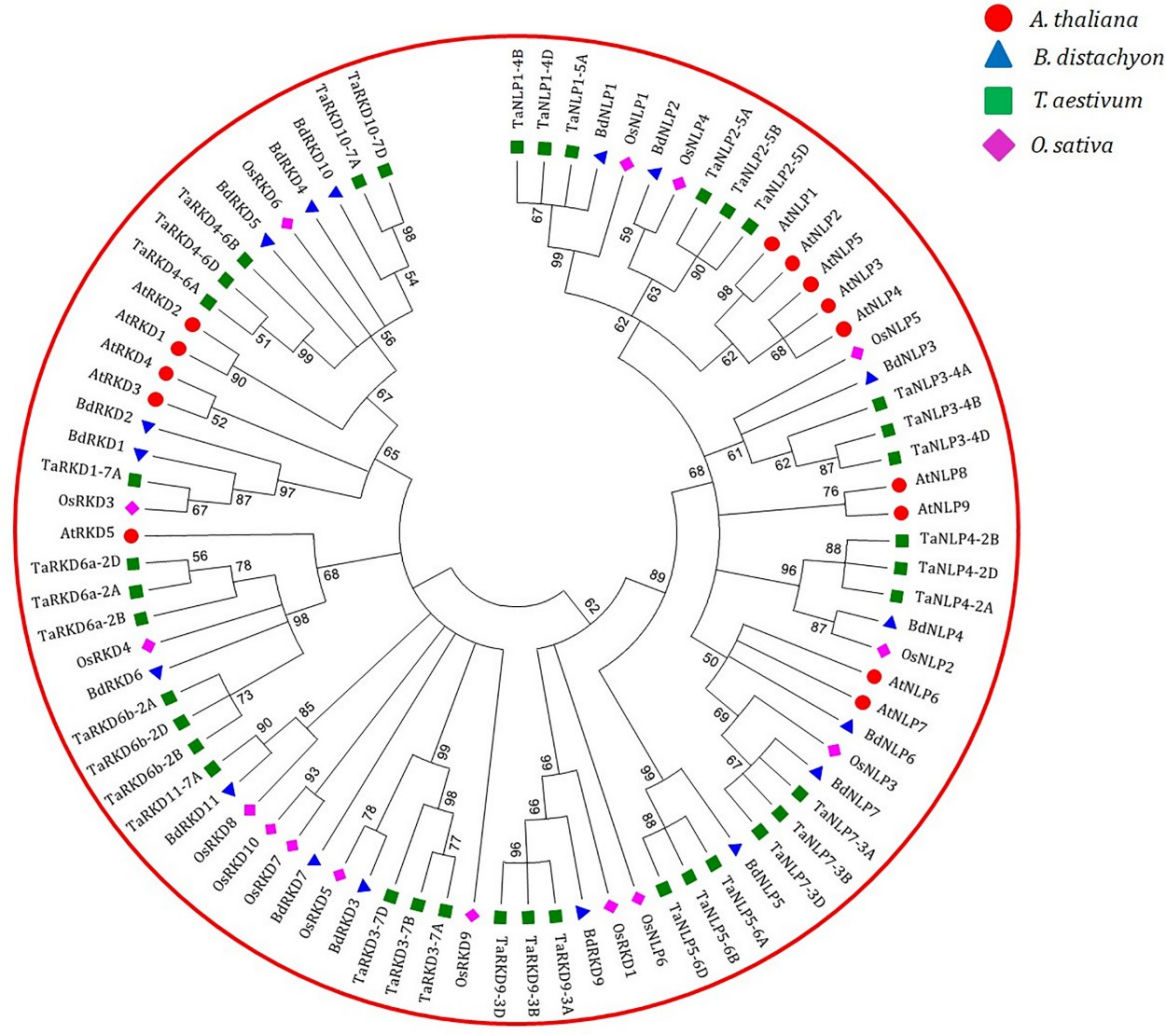
Phylogenetic tree constructed using proteins sequences of RKDs and NLPs belonging to four plant species (*A*. *thaliana*, *B*. *distachyon*, *O*. *sativa* and *T*. *aestivum*). Red, magenta, blue and green colours represent proteins sequences of RKDs and NLPs belonging to *A*. *thaliana*, *O*. *sativa*, *B*. *distachyon* and *T*. *aestivum*, respectively. The branch length represents the magnitude of genetic change.

For phylogenetic analysis, we used a total of 83 RWP-RK proteins including 37 from wheat, 17 from Brachypodium, 15 from rice and 14 from Arabidopsis. The 83 proteins made two major clusters, cluster I largely including NLPs and cluster II largely including RKDs (Fig 8). The only outliers were OsRKD1, three TaRKD9s and BdRKD9, together forming one sub-cluster and OsNLP6 forming another sub-cluster within cluster I. The inclusion of five RKD proteins with NLPs in cluster I may be attributed to evolving nature of RKDs relative to NLPs, which are conserved. A separate sub-cluster with OsNLP6 alone may be attributed to the absence of downstream half of the protein [4]. In this respect, our results partly differ from those of an earlier study, where NLPs and RKDs from six species were shown to clearly segregate in two independent clusters with no exceptions [4]. Another noticeable feature of our analysis at cluster level is that wheat proteins in both the clusters clustered with corresponding proteins of Brachypodium along with some rice proteins. As expected, all Arabidopsis proteins clustered together making separate sub-clusters in each of the two clusters. This suggested differentiation among these proteins following the divergence of monocots and dicots from a common ancestor.

### qRT-PCR expression analysis of *TaNLP* and *TaRKD* genes

**Fig 9:**
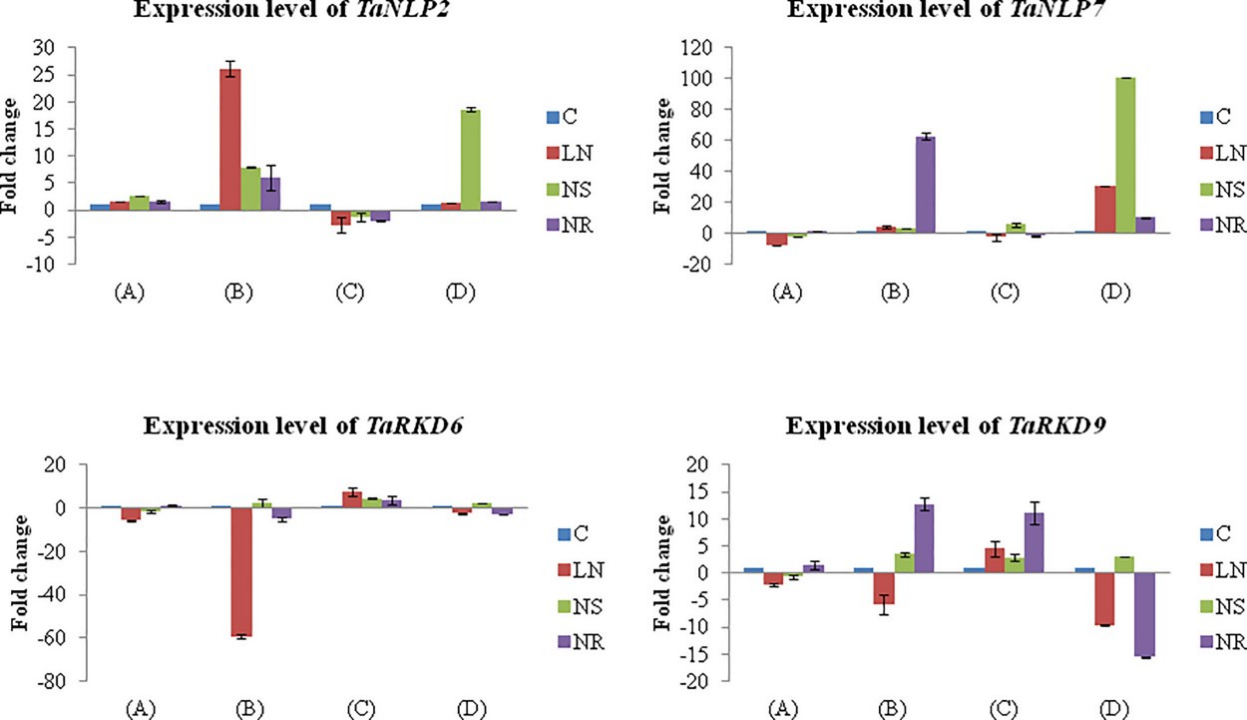
Relative expression level of two *TaNLP and* two *TaRKD* genes in root and shoot tissues of wheat seedlings. (A) C306 root, (B) C306 shoot, (C) HUW 468 root, and (D) HUW 468 shoot. Four treatments are shown by four different colours. For details of treatments, see text. C-control, LN- low N, NS- N starvation and NR- N restoration.

The four representative genes (*TaNLP2*, *TaNLP7*, *TaRKD6* and *TaRKD9*) in two wheat cultivars were used for qRT-PCR and were found to differ in expression patterns in roots and shoots under three different N regimes (Fig 9). The results differed not only for four genes, but also in two wheat genotypes, which included C306 (with low NUE) and HUW468 (with high NUE). Following is the summary of the results of qRT-PCR that are presented in Fig 9. (i) In root tissue, no major change was noticed in the expression of two *TaNLP* genes and also in *TaRKD6* gene, but ~10 fold increase in the expression of *TaRKD9* was noticed in HUW468 on N restoration; (ii) In shoot tissue, the expression of *TaNLP2* in C306 increased ~25 fold under low N, and in HUW468 it increased ~20 fold under N starvation; the expression of *TaNLP7* increased ~60 fold in C306 on N restoration, and 100 fold in HUW468 on N starvation; in shoot tissue, the expression of both *TaRKD* genes declined (-60 fold in C306 under low N in *TaRKD6*, and -15 fold in HUW468 on N restoration in *TaRKD9*).

The above summary of results suggests that the expression of two *TaNLP* genes is N-dose dependent, tissue specific and differs in two genotypes, which differ for NUE. Firstly, the expression of the two *TaNLP* genes was higher in the shoot tissues suggesting their involvement in N translocation/mobilization rather than in the uptake of N. Secondly, the expression of same genes differed under different doses of N in the two genotypes; in the genotype HUW468 with higher NUE, their expression was higher under N starvation but in C306 with low NUE, their expression was higher at low or optimum dose of N. These results clearly suggest that *TaNLP* genes may help in N translocation/mobilization in genotype HUW468 with relatively high NUE even in the absence of N, but in the genotype with low NUE, presence of N is necessary to induce these genes to help in N translocation/mobilization in the shoot.

Although the results of our qRT-PCR in seedlings cannot be compared with those of *in silico* analysis at post-anthesis stage, similar conclusions can be drawn from these two studies. For instance, *in silico* analysis of expression of two *TaNLP* genes (*TaNLP1* and *TaNLP2*) in adult leaves of the genotype Herebard, was relatively low at high dose of N (200Kg N/ha) than at a low dose of N (50 KgN/ha) suggesting their role in NUE. *NLP* genes have also been implicated in nitrate assimilation and other metabolic/regulatory processes associated with NUE [6] and *AtNLP7* has been shown to have a role in the control of the expression of nitrate transporter (*NRT1*.*1*), adaptation to limited supply of nitrogen and in signaling F-box (*AFB3*) genes in Arabidopsis [7]. However, in wheat, the role of *TaNLPs* in controlling the down-stream genes is yet to be fully understood.

The results of qRT-PCR were also subjected to ANOVA, which suggested significant variation in expression due to genes (four genes), due to tissues (root and shoot) and due to treatments including supply of different levels of N. Interactions were largely non-significant, except for the following: (i) N x V in case of *TaNLP7* in shoot, (ii) V x D in case of *TaNLP2* and *TaRKD6* in root and (iii) N x D in case of *TaNLP2* in root. This suggests that the expression of *RWP-RK* genes is controlled by several interdependent factors which form a network, and does not merely depends upon level of N supply.

*RKD* genes are known to be primarily involved in egg and sperm development, as shown in *Marchantia polymorpha* [68], Arabidopsis and wheat [69,70] and in post-zygotic embryogenesis as shown in Arabidopsis [71]. However, in our qRT-PCR analysis, *TaRKD9* was found to respond to N restoration after N starvation, suggesting a positive role of this gene in response to nitrogen signaling. The presence of nitrogen response elements in the promoter of *TaRKD3-7D* also suggests that *TaRKD* genes may be involved in response to N starvation. Therefore, the response to nitrogen status may be considered to be a novel function for at least some *TaRKD* genes, which deserves further study. However, in *Chlamydomonas reinhardii*, the change in expression level of *RKD* genes in response to N starvation is known [8], but its role in higher plants needs to be examined. In wheat also, role of *TaRKD1* and *TaRKD2* in egg development has been examined, but there is no report on a role of *RKD* genes in nitrogen signaling, although N response elements were detected in *TaRKD3-7D*. Alteration of expression of *TaRKD* genes in shoot and not in root also suggest a role of these genes in regulating the expression of many downstream genes, which may be involved in shoot development. Thus, there is a need to understand the role of the *TaRKD* genes in vegetative tissues and their control on the expression of down-stream genes in response to N application.

## Supporting information

S1 Fig Representative figure showing synteny and collinearity of wheat *TaNLP1-4D* gene with respective genes of Brachypodium, rice and sorghum. *TaNLP1-4D* gene (with a green boundary) in wheat is connected with corresponding gene in Brachypodium, rice and sorghum by a thick green line.
(TIF)

S2 Fig Results of *in silico* expression analysis, hierarchical clustering of *TaRKDs* and *TaNLPs* in different parts of a plant. (TIF)

S3 Fig Results of *in silico* expression analysis, hierarchical clustering of *TaRKDs* and *TaNLPs* under different development stages. (TIF)

S4 Fig Superimposed 3D structures of TaRKD and TaNLP proteins over 3D structures of BdRKD and BdNLP proteins (shown in grey colour). (TIF)

S5 Fig Gene ontology analysis of TaRKD and TaNLP proteins: (a) predicted biological process and, (b) predicted biochemical functions.
(TIF)

S1 Table List of primers for representative genes used in quantitative real time-PCR (qRT-PCR) expression profiling. (DOCX)

S2 Table Simple sequence repeats (SSRs) identified in *TaRKD and TaNLP* genes. (DOCX)

S3 Table The starting position of various regulatory elements from transcription start site identified in 1kb upstream promoter region of *RWP-RK* genes in wheat. (DOCX)

S4 Table Physicochemical properties of TaRKD and TaNLP proteins. (DOCX)

S5 Table Homology modeling and structure validation of TaRKD and TaNLP proteins using Swiss-Model and Protein Structure Validation Suite (PSVS) respectively, along with their PMDB accessions. (DOCX)

S6 Table Predicted values of different parameters after superimposition of 3D structures of TaRKD and TaNLP proteins over 3D structure of BdRKD and BdNLP proteins. (DOCX)

S7 Table Sub-cellular localization of TaRKD and TaNLP proteins. (DOCX)
