# Supplementary figures and images for: Genome-wide identification and characterization of gene family for RWP-RK transcription factors in wheat (Triticum aestivum L.)
Source: PLoS One. 2018 Dec 12;13(12):e0208409. doi: 10.1371/journal.pone.0208409 (PMC6291158; doi:10.1371/journal.pone.0208409)

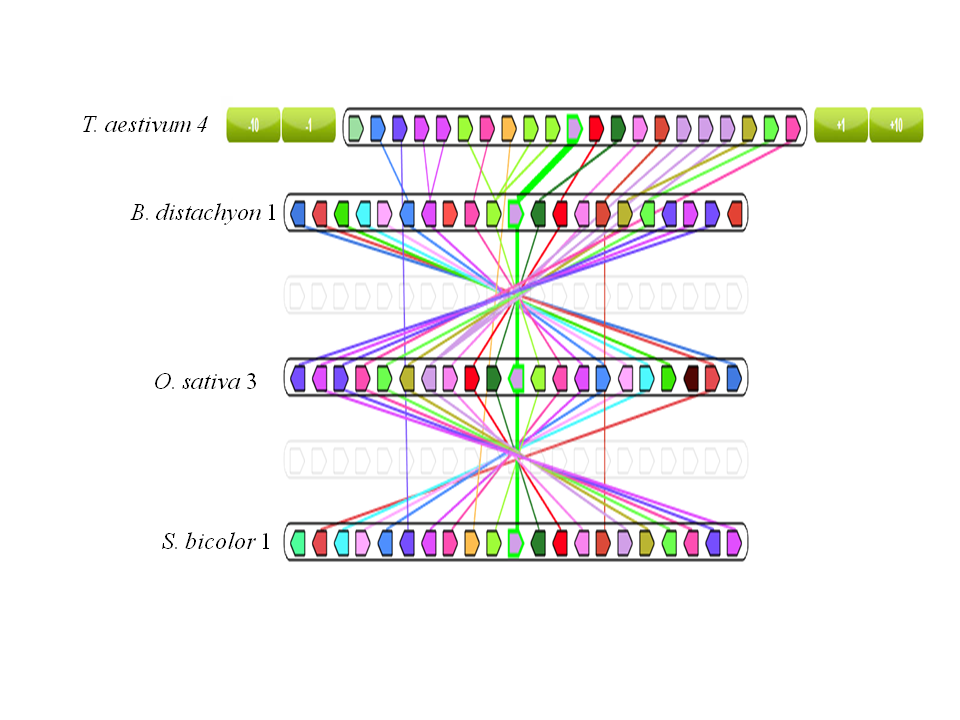

Supplement: S1 Fig — TaNLP1-4D gene (with a green boundary) in wheat is connected with corresponding gene in Brachypodium, rice and sorghum by a thick green line. (TIF) [file pone.0208409.s001.tif]

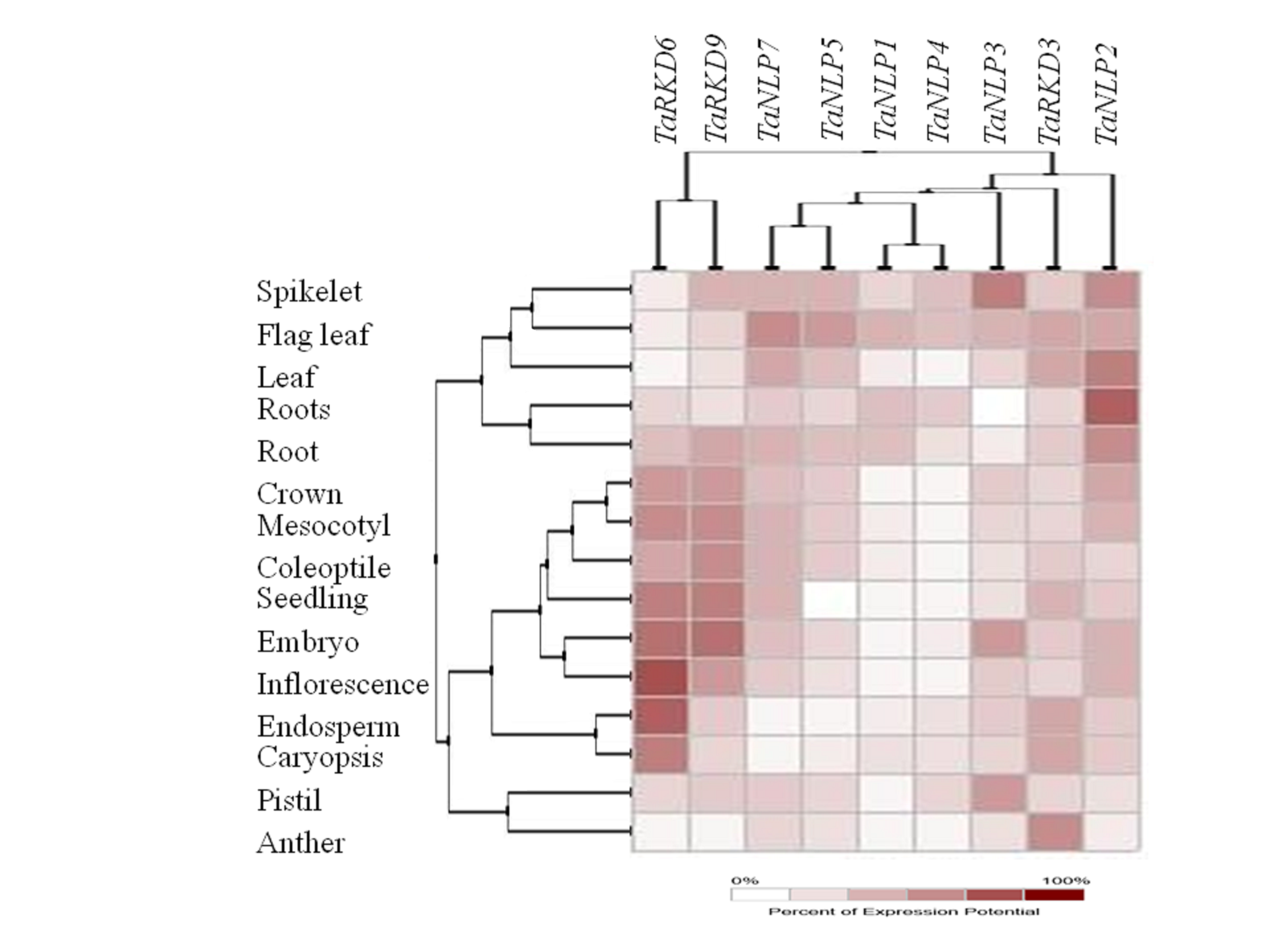

Supplement: S2 Fig — (TIF) [file pone.0208409.s002.tif]

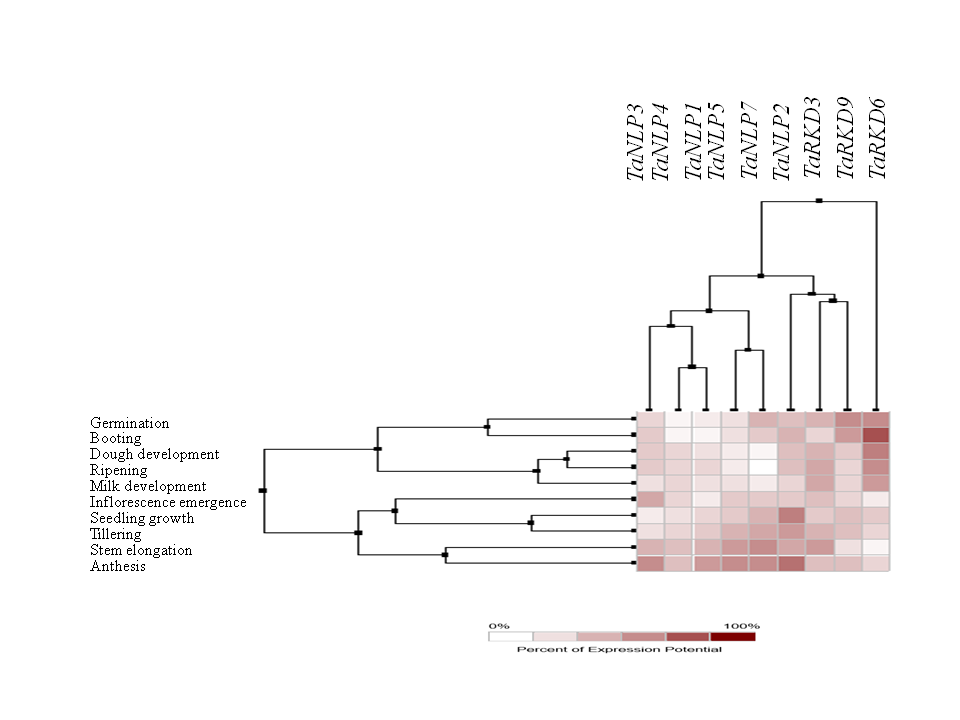

Supplement: S3 Fig — (TIF) [file pone.0208409.s003.tif]

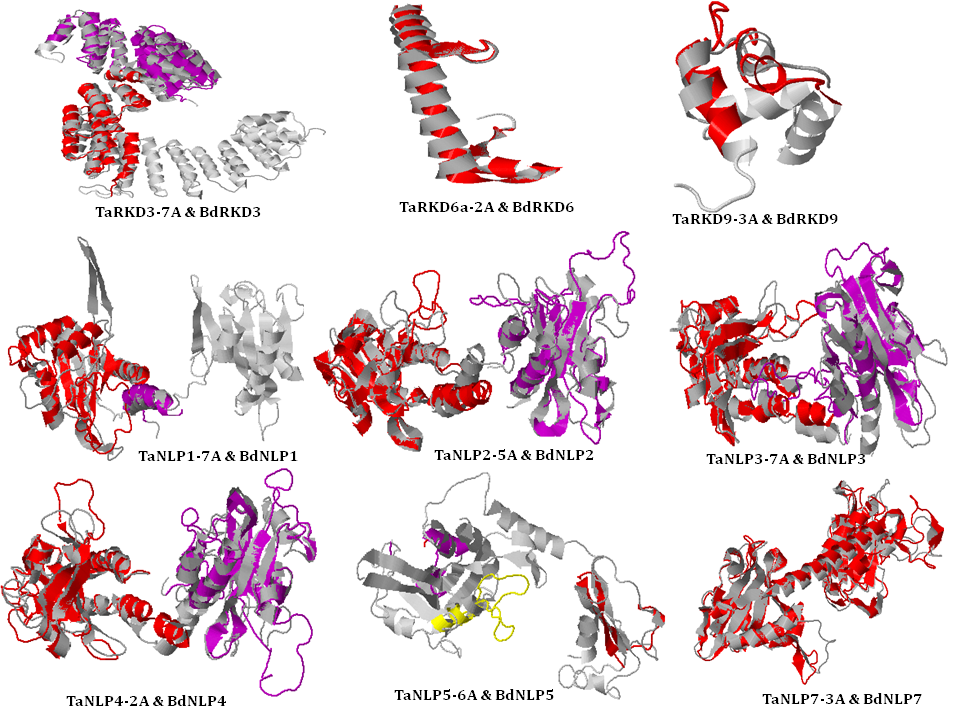

Supplement: S4 Fig — (TIF) [file pone.0208409.s004.tif]

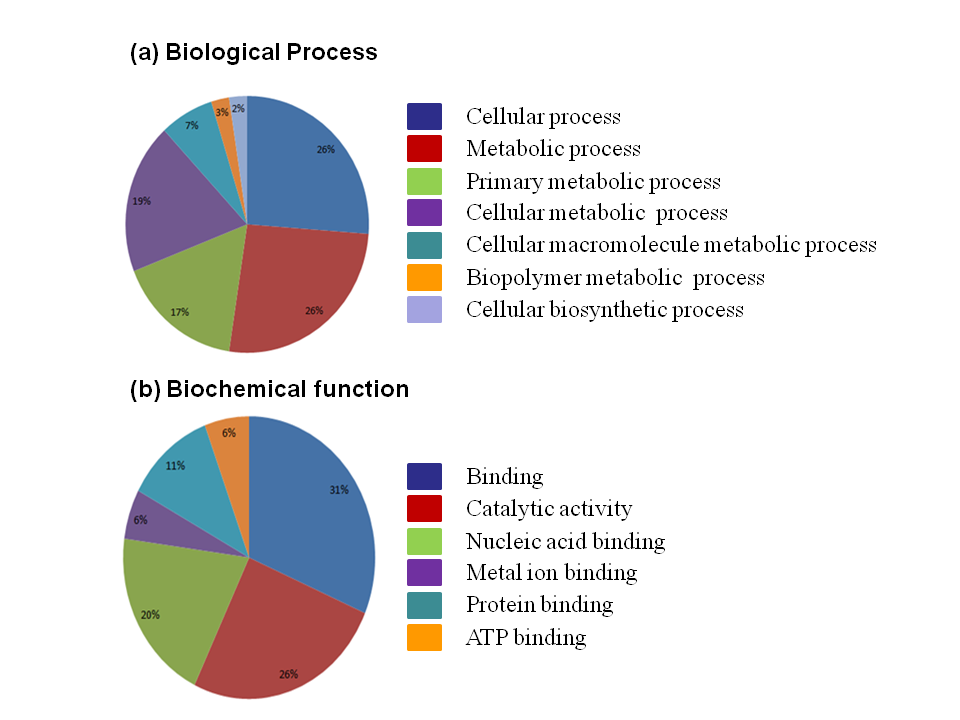

Supplement: S5 Fig — Gene ontology analysis of TaRKD and TaNLP proteins: (a) predicted biological process and, (b) predicted biochemical functions. (TIF) [file pone.0208409.s005.tif]
