# Supplementary material for: Genome-wide identification and characterization of gene family for RWP-RK transcription factors in wheat (Triticum aestivum L.)
Source: PLoS One. 2018 Dec 12;13(12):e0208409. doi: 10.1371/journal.pone.0208409 (PMC6291158; doi:10.1371/journal.pone.0208409)
Supplement: S1 Table — (DOCX) [file pone.0208409.s006.docx]

**Supplementary material**

**Genome-Wide Identification and Characterization of Gene Family for RWP-RK Transcription Factors in Wheat**

(***Triticum aestivum* L.**)

Anuj Kumar^1^*¶*, Ritu Batra^2^*¶*, Vijay Gahlaut^3^, Tinku Gautam^2^, Sanjay Kumar^4^, Mansi Sharma^5^, Sandhya Tyagi^7^, Krishna Pal Singh^1,6^, H. S. Balyan^2^ , Renu Pandey^7^, and P.K. Gupta*^2^

*Correspondence:

P.K.Gupta

Email id: pkgupta36@gmail.com

Phone: +91-[9411619105](tel:094116%2019105)

**Supplementary Table 1**. List of primers for representative genes used in quantitative real time-PCR (qRT-PCR) expression profiling.

| Gene name | Forward primers (5’-3’) | Reverse primers (5’-3’) |
| --- | --- | --- |
| *TaNLP2* | GGCAAGCTACCTGAGTGGTC | CGTCCAAATACTGTGCATGG |
|  | ACTGCATCTCCACCATTGAC | GAAGGTGGTGGTCAGAGCA |
| *TaNLP7* | GGAGCCCATGAGTTCGATAA | CGTCCAAATACTGTGCATGG |
|  | TCACCCGAAGGTGATACACA | CGTCCAAATACTGTGCATGG |
| *TaRKD6* | CCCTGTACAACGACCTGACA | GCATCCGAAGTGGTCAATTT |
|  | CCCTGTACAACGACCTGACA | CCCTGCCTCTTGCTTAACAG |
| *TaRKD9* | TTGTGCCGACTGAACAAGAG | CTTGCTCCTCCACTGCT |
