# Supplementary material for: Genome-wide identification and characterization of gene family for RWP-RK transcription factors in wheat (Triticum aestivum L.)
Source: PLoS One. 2018 Dec 12;13(12):e0208409. doi: 10.1371/journal.pone.0208409 (PMC6291158; doi:10.1371/journal.pone.0208409)
Supplement: S2 Table — (DOCX) [file pone.0208409.s007.docx]

**Supplementary material**

**Genome-Wide Identification and Characterization of Gene Family for RWP-RK Transcription Factors in Wheat**

(***Triticum aestivum* L.**)

Anuj Kumar^1^*¶*, Ritu Batra^2^*¶*, Vijay Gahlaut^3^, Tinku Gautam^2^, Sanjay Kumar^4^, Mansi Sharma^5^, Sandhya Tyagi^7^, Krishna Pal Singh^1,6^, H. S. Balyan^2^ , Renu Pandey^7^, and P.K. Gupta*^2^

*Correspondence:

P.K.Gupta

Email id: pkgupta36@gmail.com

Phone: +91-[9411619105](tel:094116%2019105)

**Supplementary Table 2**. Simple sequence repeats (SSRs) identified in *TaRKD and TaNLP* genes.

| Wheat gene | Chromosome | Count | Motif | Motif length |
| --- | --- | --- | --- | --- |
| *TaRKD3-7B* | 7B | 1 | CCGCGG | 6 |
| *TaRKD4-6B* | 6B | 1 | TAC | 3 |
| *TaRKD4-6D* | 6D | 1 | TAC | 3 |
| *TaRKD6a-2A* | 2A | 1 | CGC | 3 |
| *TaRKD6a-2B* | 2B | 1 | CGC | 3 |
| *TaRKD6a-2D* | 2D | 1 | CGC | 3 |
| *TaRKD6b-2A* | 2A | 1 | GCC | 3 |
| *TaNLP1-4B* | 4B | 1 | TACG | 4 |
|  |  | 2 | AGAGGC* | 6 |
|  |  | 1 | TCC | 3 |
|  |  | 3 | CCCCAC* | 6 |
| *TaNLP1-4D* | 4D | 1 | CGA | 3 |
| *TaNLP2-5A* | 5A | 1 | CCA | 3 |
| *TaNLP2-5D* | 5D | 1 | GAGG | 4 |
| *TaNLP4-2A* | 2A | 1 | CAGC | 4 |
| *TaNLP4-2D* | 2D | 1 | CAGC | 4 |
| *TaNLP5-6A* | 6A | 1 | GCG | 3 |
|  |  | 2 | GAA* | 3 |
| *TaNLP5-6B* | 6B | 1 | GAG | 3 |
| *TaNLP7-3A* | 3A | 1 | CCT | 3 |

A star (*) in this column means multi-locus SSR.
