# Supplementary material for: Genome-wide identification and characterization of gene family for RWP-RK transcription factors in wheat (Triticum aestivum L.)
Source: PLoS One. 2018 Dec 12;13(12):e0208409. doi: 10.1371/journal.pone.0208409 (PMC6291158; doi:10.1371/journal.pone.0208409)
Supplement: S3 Table — (DOCX) [file pone.0208409.s008.docx]

**Supplementary material**

**Genome-Wide Identification and Characterization of Gene Family for RWP-RK Transcription Factors in Wheat** (***Triticum aestivum* L.**)

Anuj Kumar^1^*¶*, Ritu Batra^2^*¶*, Vijay Gahlaut^3^, Tinku Gautam^2^, Sanjay Kumar^4^, Mansi Sharma^5^, Sandhya Tyagi^7^, Krishna Pal Singh^1,6^, H. S. Balyan^2^ , Renu Pandey^7^, and P.K. Gupta*^2^

*Correspondence:

P.K.Gupta

Email id: pkgupta36@gmail.com

Phone: +91-[9411619105](tel:094116%2019105)

**Supplementary Table 3.** The starting position of various regulatory elements from transcription start site identified in 1kb upstream promoter region of *RWP-RK* genes in wheat.

| **Gene name** | **TATA box** | **CAAT box** | **Tissue specific** | **Light responsive** | **Abiotic and biotic stress responsive** |
| --- | --- | --- | --- | --- | --- |
| *TaRKD1-*7A | 443 | 282, 769 |  | 533, 964 | 170, 372, 455, 476, 502, 552, 558, 586, 769, 841, 872, 947 |
| *TaRKD3-7A* | 100, 109, 133, 176, 178, 390, 392, 523, 805, 806, 832, 886 | 43, 66, 308, 545, 564, 619, 670, 685, 858 | 437 (E) | 225, 316, 409, 685, 694, 851, 958 | 238, 927 |
| *TaRKD3-7B* | 567, 570, 686, 723, 724, 734, 788, 873 | 698 | 46, 92, 318, 772, 837, 853 | 20, 214, 302, 388, 695 | 40, 155, 217, 302, 388, 494, 694, 878, 930 |
| *TaRKD3-7D* | 341, 390, 404, 587, 637, 681, 683, 684, 751, 776, 804, 831, 845 | - | 94 (M), 19, 63, 742, 758 (E) | 1, 162, 191, 227, 512, 768, 949, 957 | 17, 61, 185, 352, 357, 648, 662, 782, 907 |
| *TaRKD4-6A* | 110, 154 | 14, 60, 363, 542, 637 | 493 (M) | 281, 525, 631 | 14, 84, 207, 210, 262, 294, 297, 563 |
| *TaRKD4-6B* | 97, 98, 112, 113 | 19, 362, 379, 640 | 69 (S), 492 (M) | 514, 524, 634, 646 | 19, 164, 210, 261, 293, 296 |
| *TaRKD4-6D* | - | 83, 121, 359, 641 | 489 (M) | 511, 521, 635 | 8, 107, 207, 345, 559 |
| *TaRKD6a-2A** | 564 | 28, 553 | 64, 96 (M) | 115, 176, 313, 376, 834, 839, 876, 979, 989 | 9, 22, 242, 314, 323, 362, 454, 925 |
| *TaRKD6a-2B** | - | 51, 588, 992 | 87, 120 (M), 376 (E) | 93, 132, 137, 157, 158, 199, 325, 388, 729, 837, 841, 846, 988 | 32, 45, 260, 326, 335, 374, 466, 934, 955 |
| *TaRKD6a-2D** | 12, 51, 67, 793 | 64, 126, 610 | 163, 195, 993 (M) | 199, 251, 307, 375, 847, 852, 886, 979, 989 | 78, 754, 308, 376, 385, 418, 517, 941 |
| *TaRKD6b-2A* | - | 553 | - | 876 | 9, 22, 176, 242, 314, 323, 438, 454, 925 |
| *TaRKD6b-2B** | - | 588, 992 | - | 93, 729 | 32, 45, 199, 260, 326, 335, 450, 466, 934, 955 |
| *TaRKD6b-2D** | 12, 67 | 64, 610 | - | 754, 886 | 251, 308, 376, 385, 501, 517, 941 |
| *TaRKD9-3A*** | 864 | 38, 59, 127, 128, 146, 352 | 251, 905, 910 (M), 162 (E) | 26, 85, 154, 187, 262, 704, 723 | 573, 725 |
| *TaRKD9-3B*** | 308 | 516, 522, 599, 849, 989, | 548, 904, 909 (M) | 390, 435, 515, 654, 862 | 81, 109, 263, 612 |
| *TaRKD9-3D*** | 99, 324, 379, 433, 434, 435, 473, 519, 570, 583 | 234, 235, 578, 832, 974 | 887, 892 (M) | 88, 136, 189, 270, 845, 993, 994 | 136, 139, 203, 258 |
| *TaRKD10-7A* | - | 587, 783, 860 | - | 20, 147, 636 | 43, 449 |
| *TaRKD10-7D* | - | 50, 548, 807 | 125 (M) | 662, 763 | 163, 319, 431, 460, 521, 569, 719 |
| *TaRKD11-7A** | - | - | - | 224, 275, 389 | 144, 238, 575, 588, 714, 778, 816, 940 |
| *TaNLP1-5A* | - | 846 | 763 (E) | 17, 518, 976 | 232, 259, 444, 530, 540, 762 |
| *TaNLP1*-4B | - | 846 | 763 (E) | 90, 439, 444, 770, 933 | 232, 259, 444, 530, 540, 762 |
| *TaNLP1*-4D | - | 51, 720, 790 | 38, 918 (M) | 59, 65, 93, 107, 187, 516, 522, 587, 619, 624, 669 | 231, 277, 347, 637, 835 |
| *TaNLP2-5A* | 11, 13, 287, 413, 415, 448 | 22, 31, 160, 241, 424, 749, 967 | - | 183, 584, , 674, 696 | 155, 236 |
| *TaNLP2-5B* | 15, 17, 210, 291, 418, 419, 451 | 34, 164, 245, 428, 752, 967 | - | 322, 381, 587, 677, 699 | 216, 240, 444 |
| *TaNLP2-5D* | 113, 139, 149, 342, 625, 640, 649, 819, 839, 845, 846, 862, 935 | 168, 169, 511, 979 | 229 (E) | 428, 491, 658, 676 | 120, 228, 518, 654, 748 |
| *TaNLP3-4A* | 188, 269, 396, 397, 430 | 3, 12, 142, 223, 406, 725, 967 | - | 300, 359, 650, 672 | 137, 155, 218 |
| *TaNLP3-4B* | 207 | - | - | 61, 90, 338, 400 | 3, 93, 339, 355 |
| *TaNLP3-4D* | 5, 113, 372 | 893 | - | 225, 254, 303, 504, 566, 865 | 141, 167, 257, 505, 521, 641 |
| *TaNLP4-2A* | 11, 594, 626, 911 | 138, 248, 364, 979 | 309, 904 (M), 801 (E) | 32, 169, 203, 240, 247, 769 | 294, 491, 746, 749, 750 |
| *TaNLP4-2B* | 44, 652, 621, 917 | 160, 390, 608, 612, 980 | 335 (M) | 49, 65, 191, 195, 229, 266, 910 | 1, 240, 272, 326, 690, 743, 746, 747 |
| *TaNLP4-2D* | 17, 627, 595, 911 | 134, 253, 369, 979 | 314 (M), 801 (E) | 38, 170, 174, 208, 245, 252, 904 | 234, 576, 746 |
| *TaNLP5-6A* | 85, 493, 677, 806 | 18, 507, 635, 711, 834 | 103, 400 (E) | 339, 394, 395, 501, 735-767 | 27, 112, 222, 238, 395, 817, 846, 978, 985 |
| *TaNLP5-6B* | 59, 74, 227, 327 | 193, 355 | 469 (E) | 67, 634, 782, 870, 925 | 338, 367, 508, 854 |
| *TaNLP5-6D* | 475, 513, 790 | 178, 655, 818 | 502 (M) | 419, 456 | 187, 393, 441, 747, 801, 830, 977 |
| *TaNLP7-3A* | 383, 592, 630, 633, 638, 651, 762 | 19, 20, 48, 53, 55, 371, 731, 745, 930 | 307 (S) | 183, 355 | 75, 236, 575 |
| *TaNLP7-3B* | 509, 565, 794 | 247, 293, 371, 595, 655 | 766 (M) | 363, 704, 727, 846, 847, 851, 884 | 281, 456, 533, 959, 979 |
| *TaNLP7-3D* | 19, 92, 118, 144 | 211, 336, 553, 657 | - | 307, 312, 328, 728 | 135, 246, 258, 307, 308, 415, 491, 839, 959 |

- response elements absent; *response element for zein regulatory metabolism present; **response element for circadian rhythm present; M- meristem; E- endosperm; S- shoot
