# Supplementary material for: Genome-wide identification and characterization of gene family for RWP-RK transcription factors in wheat (Triticum aestivum L.)
Source: PLoS One. 2018 Dec 12;13(12):e0208409. doi: 10.1371/journal.pone.0208409 (PMC6291158; doi:10.1371/journal.pone.0208409)
Supplement: S4 Table — (DOCX) [file pone.0208409.s009.docx]

**Supplementary material**

**Genome-Wide Identification and Characterization of Gene Family for RWP-RK Transcription Factors in Wheat**

(***Triticum aestivum* L.**)

Anuj Kumar^1^*¶*, Ritu Batra^2^*¶*, Vijay Gahlaut^3^, Tinku Gautam^2^, Sanjay Kumar^4^, Mansi Sharma^5^, Sandhya Tyagi^7^, Krishna Pal Singh^1,6^, H. S. Balyan^2^ , Renu Pandey^7^, and P.K. Gupta*^2^

*Correspondence:

P.K.Gupta

Email id: pkgupta36@gmail.com

Phone: +91-[9411619105](tel:094116%2019105)

**Supplementary Table 4**. Physicochemical properties of TaRKD and TaNLP proteins.

| **Protein identity** | **M.wt (KD)** | **pI** | **Ins.I** | **AI** | **Gravy** |
| --- | --- | --- | --- | --- | --- |
| TaRKD1-7A | 38.40 | 5.07 | 61.37 | 75.78 | -0.414 |
| TaRKD3-7A | 75.79 | 7.38 | 43.19 | 81.31 | -0.153 |
| TaRKD3-7B | 103.68 | 6.84 | 48.27 | 81.21 | -0.247 |
| TaRKD3-7D | 75.73 | 6.95 | 43.82 | 81.47 | -0.159 |
| TaRKD4-6A | 23.85 | 8.49 | 64.32 | 84.17 | -0.629 |
| TaRKD4-6B | 23.75 | 8.23 | 60.31 | 86.52 | -0.600 |
| TaRKD4-6D | 23.78 | 9.12 | 63.00 | 86.10 | -0.549 |
| TaRKD6a-2A | 39.37 | 9.08 | 52.84 | 78.93 | -0.612 |
| TaRKD6a-2B | 40.96 | 9.80 | 52.15 | 81.13 | -0.439 |
| TaRKD6a-2D | 45.46 | 8.70 | 52.31 | 84.55 | -0.417 |
| TaRKD6b-2A | 38.10 | 8.42 | 40.98 | 80.24 | -0.491 |
| TaRKD6b-2B | UI | UI | 40.54 | 77.95 | -0.514 |
| TaRKD6b-2D | 38.04 | 8.61 | 40.86 | 79.38 | -0.510 |
| TaRKD9-3A | 32.60 | 8.80 | 46.79 | 71.63 | -0.527 |
| TaRKD9-3B | 32.13 | 8.27 | 42.42 | 72.16 | -0.487 |
| TaRKD9-3D | 32.29 | 8.58 | 44.00 | 71.92 | -0.526 |
| TaRKD10-7A | 28.35 | 5.13 | 66.35 | 81.81 | -0.497 |
| TaRKD10-7D | 29.20 | 5.07 | 58.57 | 82.30 | -0.464 |
| TaRKD11-7A | 40.52 | 5.54 | 49.98 | 72.71 | -0.498 |
| TaNLP1-5A | 99.97 | 5.56 | 50.82 | 72.25 | -0.468 |
| TaNLP1-4B | 100.17 | 5.63 | 53.94 | 73.58 | -0.446 |
| TaNLP1-4D | 99.85 | 5.70 | 51.07 | 73.20 | -0.459 |
| TaNLP2-5A | 98.65 | 6.59 | 51.06 | 71.71 | -0.469 |
| TaNLP2-5B | 98.68 | 6.45 | 50.67 | 73.42 | -0.430 |
| TaNLP2-5D | 98.76 | 6.86 | 48.27 | 73.31 | -0.459 |
| TaNLP3-4A | 98.56 | 5.42 | 46.65 | 79.97 | -0.319 |
| TaNLP3-4B | 100.32 | 5.42 | 47.86 | 79.28 | -0.342 |
| TaNLP3-4D | 100.10 | 5.43 | 46.02 | 79.91 | -0.334 |
| TaNLP4-2A | 102.04 | 5.76 | 51.07 | 71.64 | -0.426 |
| TaNLP4-2B | 102.11 | 5.75 | 51.35 | 71.02 | -0.441 |
| TaNLP4-2D | 102.06 | 5.72 | 49.95 | 71.22 | -0.435 |
| TaNLP5-6A | 77.76 | 5.78 | 49.59 | 76.41 | -0.315 |
| TaNLP5-6B | 59.70 | 5.75 | 52.88 | 79.00 | -0.273 |
| TaNLP5-6D | 77.31 | 5.97 | 51.54 | 76.27 | -0.353 |
| TaNLP7-3A | 102.31 | 5.71 | 51.95 | 75.04 | -0.377 |
| TaNLP7-3B | 102.24 | 5.82 | 52.26 | 75.25 | -0.367 |
| TaNLP7-3D | 102.24 | 5.71 | 51.71 | 75.04 | -0.371 |

M.wt - Molecular weight, pI - isoelectric point, Ins.I - Instability index, AI - Aliphatic index, Gravy - Grand average of hydropathy, UI- unidentified
