# Supplementary material for: Genome-wide identification and characterization of gene family for RWP-RK transcription factors in wheat (Triticum aestivum L.)
Source: PLoS One. 2018 Dec 12;13(12):e0208409. doi: 10.1371/journal.pone.0208409 (PMC6291158; doi:10.1371/journal.pone.0208409)
Supplement: S5 Table — (DOCX) [file pone.0208409.s010.docx]

**Supplementary material**

**Genome-Wide Identification and Characterization of Gene Family for RWP-RK Transcription Factors in Wheat**

(***Triticum aestivum* L.**)

Anuj Kumar^1^*¶*, Ritu Batra^2^*¶*, Vijay Gahlaut^3^, Tinku Gautam^2^, Sanjay Kumar^4^, Mansi Sharma^5^, Sandhya Tyagi^7^, Krishna Pal Singh^1,6^, H. S. Balyan^2^ , Renu Pandey^7^, and P.K. Gupta*^2^

*Correspondence:

P.K.Gupta

Email id: pkgupta36@gmail.com

Phone: +91-[9411619105](tel:094116%2019105)

**Supplementary Table 5.** Homology modeling and structure validation of TaRKD and TaNLP proteins using Swiss-Model and Protein Structure Validation Suite (PSVS) respectively, along with their PMDB accessions.

| **Homology modeling** | | | | | **Structure validation** | **PMDB Id** |
| --- | --- | --- | --- | --- | --- | --- |
| **Protein** | **Template PDB ID** | **Template description** | **Chain** | **Model oligo-state** | **Ramachandran plot**  **(Statistics)** |  |
| TaRKD1-7A | [3oq9.1](https://swissmodel.expasy.org/templates/3oq9.1) | Protein FADD | F | Monomer | Favoured region: 82.0%,  Allowed region: 15.3%,  Generously region: 1.8%,  Disallowed region: 0.9% | PM0081797 |
| TaRKD3-7A | 4m57.1 | Chloroplast pentatricopeptide  repeat protein 10 | A | Monomer | Favoured region: 89.6%,  Allowed region: 8.3%,  Generously region: 1.2%,  Disallowed region: 0.9% | PM0079803 |
| TaRKD4-6A | [4ihw.1.B](https://swissmodel.expasy.org/templates/4ihw.1) | DNA-binding protein fis | B | Homo-dimer | Favoured region: 83.3%,  Allowed region: 8.0%,  Generously region: 7.3%,  Disallowed region: 1.3% | PM0081798 |
| TaRKD6a-2A | 1s7o.1 | Hypothetical UPF0122 protein  SPy1201/SpyM3_0842/SPs1042/spyM18_1152 | C | Monomer | Favoured region: 96.3%,  Allowed region: 3.7%,  Generously region: 0.0 %,  Disallowed region: 0.0% | PM0079805 |
| TaRKD9-3A | 2ziw.1 | Mus81 protein | A | Monomer | Favoured region: 78.3%,  Allowed region: 8.7%,  Generously region: 6.5%,  Disallowed region: 6.5% | PM0079806 |
| TaRKD10-7A | [1zx4.1](https://swissmodel.expasy.org/templates/1zx4.1) | Plasmid Partition par B protein | C | Homo-dimer | Favoured region: 88.7%,  Allowed region: 8.8%,  Generously region: 2.0%,  Disallowed region: 0.5% | PM0081799 |
| TaRKD11-7A | [5mrc.36](https://swissmodel.expasy.org/templates/5mrc.36) | mL58 | A | Monomer | Favoured region: 95.8%,  Allowed region: 4.2%,  Generously region: 0.0%,  Disallowed region: 0.0% | PM0081800 |
| TaNLP1-4B | 3ix8.1 | Transcriptional activator protein lasR | A | Monomer | Favoured region: 81.8%,  Allowed region: 8.6%,  Generously region: 2.7%,  Disallowed region: 1.4% | PM0079796 |
| TaNLP2-5A | 3ibj.1 | cGMP-dependent 3',5'-cyclicphosphodiesterase | B | Monomer | Favoured region:81.8%,  Allowed region: 13.6%,  Generously region: 2.6%,  Disallowed region: 2.0% | PM0079797 |
| TaNLP3-4A | 3ibj.1 | cGMP-dependent 3',5'-cyclicphosphodiesterase | A | Monomer | Favoured region: 84.7%,  Allowed region: 10.4%,  Generously region: 3.3%,  Disallowed region: 1.6% | PM0079798 |
| TaNLP4-2A | 3mf0.1 | cGMP-specific 3',5'-cyclic  phosphodiesterase | A | Monomer | Favoured region: 81.1%,  Allowed region:12.5 %,  Generously region:4.0 %,  Disallowed region: 2.4% | PM0079799 |
| TaNLP5-6A | 1wj6.1 | KIAA0049 protein | A | Monomer | Favoured region: 75.9%,  Allowed region: 20.5%,  Generously region: 1.2%,  Disallowed region: 2.4% | PM0079800 |
| TaNLP7-3A | 3ibj.1 | cGMP-dependent 3',5'-cyclicphosphodiesterase | A | Monomer | Favoured region: 80.3%,  Allowed region: 14.9%,  Generously region: 2.3%,  Disallowed region: 2.6% | PM0079801 |
