# Supplementary material for: Genome-wide identification and characterization of gene family for RWP-RK transcription factors in wheat (Triticum aestivum L.)
Source: PLoS One. 2018 Dec 12;13(12):e0208409. doi: 10.1371/journal.pone.0208409 (PMC6291158; doi:10.1371/journal.pone.0208409)
Supplement: S6 Table — (DOCX) [file pone.0208409.s011.docx]

**Supplementary material**

**Genome-Wide Identification and Characterization of Gene Family for RWP-RK Transcription Factors in Wheat**

(***Triticum aestivum* L.**)

Anuj Kumar^1^*¶*, Ritu Batra^2^*¶*, Vijay Gahlaut^3^, Tinku Gautam^2^, Sanjay Kumar^4^, Mansi Sharma^5^, Sandhya Tyagi^7^, Krishna Pal Singh^1,6^, H. S. Balyan^2^ , Renu Pandey^7^, and P.K. Gupta*^2^

*Correspondence:

P.K.Gupta

Email id: pkgupta36@gmail.com

Phone: +91-[9411619105](tel:094116%2019105)

**Supplementary Table 6.** Predicted values of different parameters after superimposition of 3D structures of TaRKD and TaNLP proteins over 3D structure of BdRKD and BdNLP proteins.

| S.NO | Pairs of proteins | Similarity (%) | P-value | RMSD | No. of twists | *Significant similarity (Yes/No) |
| --- | --- | --- | --- | --- | --- | --- |
| 1. | TaRKD1-7A & BdRKD1 | 25.30 | 4.58e-02 | 2.73 | 1 | Yes |
| 2. | TaRKD3-7A & BdRKD3 | 36.97 | 4.53e-04 | 3.13 | 0 | Yes |
| 3. | TaRKD4-6A & BdRKD4 | 91.53 | 5.58e-14 | 0.97 | 0 | No |
| 4. | TaRKD6a-2A & BdRKD6 | 88.14 | 0.00e+00 | 0.11 | 0 | Yes |
| 5. | TaRKD9-3A & BdRKD9 | 8.82 | 3.91e-01 | 3.09 | 0 | No |
| 6. | TaRKD10-7A & BdRKD10 | 29.63 | 1.07e-01 | 2.63 | 1 | No |
| 7. | TaRKD11-7A & BdRKD11 | 50.00 | 6.20e-03 | 1.83 | 1 | Yes |
| 8. | TaNLP1-4B & BdNLP1 | 20.00 | 2.55e-02 | 2.88 | 1 | Yes |
| 9. | TaNLP2-5A & BdNLP2 | 76.00 | 3.04e-13 | 3.02 | 1 | Yes |
| 10. | TaNLP3-4A & BdNLP3 | 76.76 | 6.95e-13 | 3.17 | 1 | Yes |
| 11. | TaNLP4-2A & BdNLP4 | 75.77 | 3.31e-12 | 3.05 | 1 | Yes |
| 12. | TaNLP5-6A & BdNLP5 | 8.99 | 8.55e-01 | 6.94 | 2 | No |
| 13. | TaNLP7-3A & BdNLP7 | 95.99 | 0.00e+00 | 3.09 | 0 | Yes |

* Structure pairs with probability < 0.05 are significantly similar.
