# Supplementary material for: Genome-wide identification and characterization of gene family for RWP-RK transcription factors in wheat (Triticum aestivum L.)
Source: PLoS One. 2018 Dec 12;13(12):e0208409. doi: 10.1371/journal.pone.0208409 (PMC6291158; doi:10.1371/journal.pone.0208409)
Supplement: S7 Table — (DOCX) [file pone.0208409.s012.docx]

**Supplementary material**

**Genome-Wide Identification and Characterization of Gene Family for RWP-RK Transcription Factors in Wheat**

(***Triticum aestivum* L.**)

Anuj Kumar^1^*¶*, Ritu Batra^2^*¶*, Vijay Gahlaut^3^, Tinku Gautam^2^, Sanjay Kumar^4^, Mansi Sharma^5^, Sandhya Tyagi^7^, Krishna Pal Singh^1,6^, H. S. Balyan^2^ , Renu Pandey^7^, and P.K. Gupta*^2^

*Correspondence:

P.K.Gupta

Email id: pkgupta36@gmail.com

Phone: +91-[9411619105](tel:094116%2019105)

**Supplementary Table 7**. Sub-cellular localization of TaRKD and TaNLP proteins.

| **Sub-cellular location** | **Protein identity** |
| --- | --- |
| Nucleus | TaRKD1-7A, TaRKD4-6A, TaRKD4-6B, TaRKD4-6D, TaRKD6a-2A, TaRKD6a-2B, TaRKD6a-2D, TaRKD6b-2A, TaRKD6b-2D, TaRKD9-3A, TaRKD9-3B, TaRKD9-3D, TaRKD10-7A, TaRKD10-7D, TaRKD11-7A, TaNLP1-5A, TaNLP1-4B, TaNLP1-4D, TaNLP2-5A, TaNLP2-5B, TaNLP2-5D, TaNLP3-4A, TaNLP3-4B, TaNLP3-4D, TaNLP4-2A, TaNLP4-2B, TaNLP4-2D, TaNLP5-6A, TaNLP5-6B, TaNLP5-6D, TaNLP7-3A, TaNLP7-3B, TaNLP7-3D |
| Mitochondrion | TaRKD3-7A, TaRKD3-7B, TaRKD3-7D |
| Chloroplast | TaRKD6b-2B |
